# Analysis of Active and Inactive X Chromosome Architecture Reveals the Independent Organization of 30 nm and Large-Scale Chromatin Structures

**DOI:** 10.1016/j.molcel.2010.10.013

**Published:** 2010-11-12

**Authors:** Catherine Naughton, Duncan Sproul, Charlotte Hamilton, Nick Gilbert

**Affiliations:** 1 Institute of Genetics and Molecular Medicine, The University of Edinburgh, Edinburgh EH4 2XR, UK; 2 Breakthrough Research Unit, Institute of Genetics and Molecular Medicine, The University of Edinburgh, Edinburgh EH4 2XU, UK

## Abstract

Using a genetic model, we present a high-resolution chromatin fiber analysis of transcriptionally active (Xa) and inactive (Xi) X chromosomes packaged into euchromatin and facultative heterochromatin. Our results show that gene promoters have an open chromatin structure that is enhanced upon transcriptional activation but the Xa and the Xi have similar overall 30 nm chromatin fiber structures. Therefore, the formation of facultative heterochromatin is dependent on factors that act at a level above the 30 nm fiber and transcription does not alter bulk chromatin fiber structures. However, large-scale chromatin structures on Xa are decondensed compared with the Xi and transcription inhibition is sufficient to promote large-scale chromatin compaction. We show a link between transcription and large-scale chromatin packaging independent of the bulk 30 nm chromatin fiber and propose that transcription, not the global compaction of 30 nm chromatin fibers, determines the cytological appearance of large-scale chromatin structures.

## Graphical abstract

**Graphical Abstract:**

**Figure:**
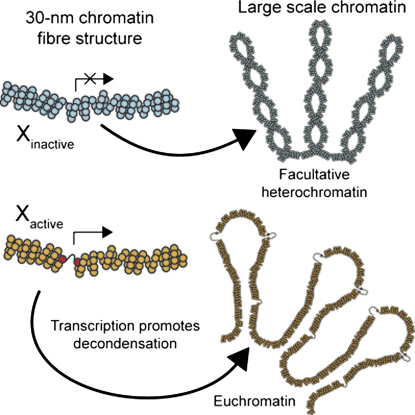

**Highlights:**

► High-resolution chromatin analysis of active and inactive X chromosomes ► Gene promoters have a transcription-dependent open chromatin structure ► Active and inactive X chromosomes have similar bulk 30 nm chromatin fiber structures ► Transcription promotes the decompaction of large-scale chromatin structures

## Introduction

Chromatin structure modulation is central to the control of gene expression (Cairns, 2009; Li et al., 2007). This is best characterized at the level of the nucleosome where histone modifications (e.g., acetylation or methylation) or histone variants have been correlated to either active transcription or gene repression (Campos and Reinberg, 2009). In cells, nucleosome arrays fold to form 30 nm chromatin fibers (Kruithof et al., 2009; Robinson and Rhodes, 2006) that can be visualized by low-angle X-ray scattering studies (Langmore and Paulson, 1983). Genome-wide mapping shows that chromatin fibers are heterogeneous with gene-rich regions being enriched in “open” chromatin (Gilbert et al., 2004) while constitutive heterochromatin has a “closed” structure (Gilbert and Allan, 2001). Open chromatin corresponds to canonical 30 nm fibers interspersed with discontinuities, probably caused by irregular nucleosome packaging. Despite a common perception that transcriptionally active and silent regions have open and closed chromatin structures, respectively, transcription is not solely dependent on structure as active genes are located in both open and closed chromatin environments (Gilbert et al., 2004). Binding of transcription factors to promoters can be occluded by nucleosomes (Fuda et al., 2009), but the formation of nuclease sensitive regions by proteins can facilitate transcription machinery access to the underlying DNA (Henikoff, 2008). Disruptions can also be introduced by chromatin remodeling machines (Clapier and Cairns, 2009) that alter the position of nucleosomes or generate regular nucleosome arrays which can be packaged into more stable chromatin structures. Although elongating RNA polymerase can read through regions of DNA packaged into nucleosomes when facilitated by other protein complexes (Li et al., 2007; Fuda et al., 2009), this process disrupts the 30 nm chromatin fiber (Shivaswamy et al., 2008), which has to be rapidly repackaged after transcription (Sapojnikova et al., 2009).

Using microscopy, a large proportion of the mammalian genome can be seen to be packaged at levels beyond the 30 nm chromatin fiber, sometimes visualized as 60–130 nm “chromonema” fibers (Belmont and Bruce, 1994). These are further folded to form large-scale chromatin structures, seen in the nucleus as euchromatin and heterochromatin. Facultative heterochromatin is chromosomal material that can adopt either a heterochromatic or euchromatic configuration depending on location (Trojer and Reinberg, 2007). By electron microscopy (EM), the facultative heterochromatin on the Xi is distinct and appears to have a loose packing of lace-like heterochromatin fibers but by DNA intercalating agents or EM, it has a more compact structure than the surrounding euchromatin (Rego et al., 2008). At the DNA sequence level, facultative heterochromatin is not characterized by repetitive sequences so is different from constitutive heterochromatin. However, facultative heterochromatin has many of the same molecular signatures as constitutive heterochromatin at the nucleosomal level, including DNA hypermethylation, histone hypoacetylation, and late replication (Richards and Elgin, 2002; Trojer and Reinberg, 2007). Like constitutive heterochromatin, it is transcriptionally inactive and is assumed to have a closed structure at the level of the fundamental 30 nm chromatin fiber (Trojer and Reinberg, 2007). However, the molecular basis for the cytological differences between active euchromatin and inactive heterochromatin has not been established.

Studies show that transcription correlates with the reorganization of large-scale chromatin structures, for example, at the level of chromosome territories transcriptionally inactive gene-poor chromosome 18 has a more compact organization that the transcriptionally active gene-rich chromosome 19 (Croft et al., 1999). Chromosomal reporters have enabled the direct visualization of genomic loci by light microscopy and showed chromatin unfolding and decondensation when transcriptional regulators are targeted to the locus (Muller et al., 2001; Tsukamoto et al., 2000; Tumbar et al., 1999). Also, decondensation of the endogenous murine HoxB locus to a structure similar to the 30 nm fiber has also been shown to accompany the induction of transcription (Chambeyron and Bickmore, 2004), indicating that large-scale chromatin structures are formed from 30 nm chromatin fibers. Furthermore, regions of open chromatin, at the level of the 30 nm fiber, are cytologically decondensed (Gilbert et al., 2004). This enabled us to suggest that chromatin fiber structures can impact on additional levels of chromatin condensation creating an environment that facilitates transcriptional activation; however, the processes linking 30 nm to higher levels of chromatin folding are unknown.

To understand the relationship between chromatin structure and transcription the chromatin fiber needs to be characterized at active and inactive genes. As chemical inhibition of transcription will directly influence the 30 nm chromatin fiber structure, we have utilized the transcriptionally active and inactive X chromosomes as a genetic model. We present a genome-wide analysis of 30 nm chromatin fiber structures using a biophysical technique (Gilbert and Allan, 2001; Gilbert et al., 2004) in conjunction with SNP arrays enabling us to contrast the structure of homologous X chromosomes. We also used 3D DNA-RNA FISH to directly investigate the compaction of large-scale chromatin structures. Despite the chromatin fiber being continuous between lower and higher levels of organization, our results reveal a novel link between transcription and large-scale chromatin packaging independent of the 30 nm chromatin fiber and propose that large-scale chromatin interactions can influence transcriptional potential by disrupting the 30 nm chromatin fiber around promoters. We also suggest it is the lack of transcription that promotes cytological chromatin condensation, not global compaction of 30 nm fibers.

## Results

### Gene Promoters Have an Open Chromatin Structure

Gene-rich regions of the human genome are enriched in “open” (disrupted) 30 nm chromatin fibers (Gilbert et al., 2004). To map these 30 nm chromatin fibers at high resolution, we developed our previously described approach to fractionate chromatin fibers based on their secondary structure and hybridized them to Illumina SNP arrays. Nuclei were prepared from SATO3 lymphoblastoid cells and digested briefly with micrococcal nuclease leaving chromatin fragments of approximately 10–20 kb. The nuclei were gently lysed and soluble chromatin was collected. To separate 30 nm chromatin fragments based on their conformation, we centrifuged them in a continuous sucrose gradient (6%–40%) under physiological salt conditions (80 mM NaCl). Under these conditions chromatin adopts a 30 nm structure (Thoma et al., 1979) and EM of chromatin from single fractions from a sucrose gradient shows it has a canonical 30 nm chromatin fiber structure (data not shown). The rationale for this fractionation approach is that if there are two chromatin fragments of the same size (and therefore mass) they will sediment together, if they have the same structure. However, if one has a more open structure it will sediment more slowly than the other due to having an increased frictional coefficient. As the chromatin released from the nuclei consists of a panoply of different sized fragments a single fraction from the sucrose gradient will contain different sized chromatin fibers having different structures. Therefore, if DNA is isolated from a single chromatin fraction from the sucrose gradient and size selected on an agarose gel DNA fragments larger than the bulk of the population will have been derived from “open” chromatin fibers while DNA fragments smaller than the bulk population will have derived from “compact” chromatin fibers. We can therefore isolate chromatin fragments that have a more “open” conformation, and sediment more slowly than would be expected for their size (and mass), and label these for hybridization to microarrays (Gilbert et al., 2004).

**Figure 1:**
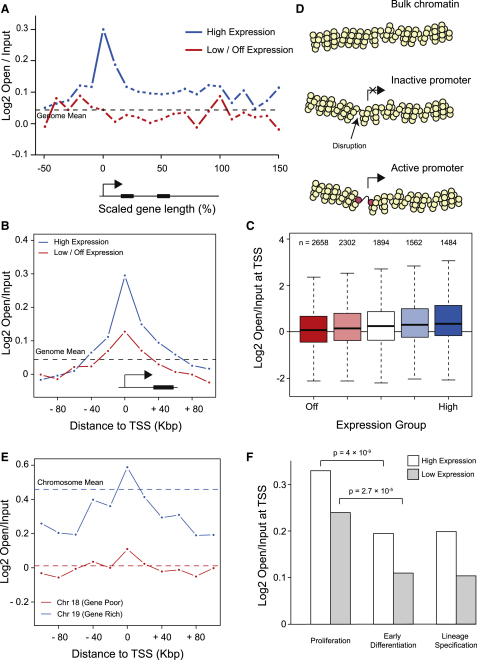
Gene Promoters Are Enriched in Open Chromatin Chromatin extracted from SATO3 cells was fractionated by sucrose gradient sedimentation and gel electrophoresis to isolate DNA probes originating from open chromatin fibers. The open chromatin probes were hybridized to Illumina SNP arrays and compared with unfractionated (input) chromatin controls. (A) Chromatin structure mapped across the scaled transcription unit of transcriptionally active and inactive genes. (B) The enrichment of open chromatin fibers in 20 kbp windows around active and inactive transcription start sites (determined from Ensembl 54). (C) The level of gene expression related to the magnitude of open chromatin at transcription start sites (2 kbp window) (n = number of SNPs analyzed). (D) Chromatin fiber modeling to predict the structure around transcription start sites (see text and Figures S1H–S1M for more details). (E) Distribution of open chromatin around the transcription start sites of genes located on HSA18 and HSA19 in 20 kbp windows. (F) Enrichment of open chromatin at the TSS of different classes of transcriptionally active and inactive genes. See also Figure S1.

Open chromatin probes were isolated as described above and hybridized to high-resolution Illumina SNP arrays. As shown previously, there is a close correlation between gene density and enrichment for open chromatin on a genome-wide level (Pearson's r = 0.84) (Figures S1A and S1B). When all genes are aligned with respect to their transcription start sites and normalized to gene length, we found that transcription start sites (TSS) of active genes are associated with a pronounced opening compared with inactive genes (Figure 1A). In addition, the body of these active genes is disrupted, possibly by irregular nucleosome positioning as a consequence of transcription (Schwabish and Struhl, 2007). Gene promoters are frequently associated with nuclease hypersensitive sensitive sites (Boyle et al., 2008) and are marked by histone modifications (Barski et al., 2007). To investigate whether the open chromatin directly corresponds to these other well known features of promoters we analyzed open chromatin in 20 kb windows around the TSS (Figure 1B). The asymmetric peak of open chromatin at promoters is broader than seen for DNaseI hypersensitive sites (Figure S1) (Boyle et al., 2008) or the distribution of the variant H2A.Z nucleosome (Barski et al., 2007) (data not shown). Surprisingly, transcriptionally inactive genes were also found to have a disruption in the chromatin fiber (Figure 1B) and the magnitude of the disruption increased with expression level (p < 1 × 10^5^ by random permutation) (Figure 1C). Although it is impossible to determine the exact structures adopted by different chromatin fibers, we have applied our modeling approach (Gilbert and Allan, 2001) on our experimental data to predict possible chromatin structures found at the promoters of active and inactive genes (Figures 1D and S1H–S1M). We suggest that bulk chromatin has one disruption every 11 nucleosomes, while inactive promoters have an additional small disruption in contrast to active promoters that have an additional large disruption.

As gene-rich chromosomes (HSA17, 19, 20) are very enriched in open chromatin (Figures S1A and S1B), we hypothesized that open chromatin might spread disrupting the chromatin of adjacent genes, creating domains of open chromatin. We therefore expected the chromatin surrounding genes on transcriptionally active and inactive chromosomes to be different. We mapped the distribution of open chromatin on the gene-rich chromosome 19 (HSA19) and the gene poor chromosome 18 (HSA18) (Figure 1E). Both chromosomes have the same proportion of transcriptionally active genes but HSA19 has on average one gene every 40 kb, while HSA18 has one gene every 160 kb, so HSA19 is more transcriptionally active. Mapping chromatin structure around the promoters of HSA18 shows that disrupted chromatin is restricted to the promoter regions but surrounding chromatin on HSA19 shows that open chromatin has spread far from promoters to more distant chromosomal regions. Mapping the chromatin structure of specific classes of genes (Zhao et al., 2007) shows that proliferation (housekeeping) genes are enriched in regions of open chromatin while regulated genes are clearly depleted in open chromatin (Figure 1F). As separate classes of genes are differently associated with open chromatin, we suggest that genes are preferentially located in specific chromatin environments (Gilbert et al., 2004). Interestingly, mapping the chromatin structure of proliferation genes on HSA19 shows that they are in a substantially more open chromatin environment than proliferation genes in general (Figures S1F and S1G). It is therefore possible that proliferation genes found on HSA19 have a requirement to be in extremely disrupted chromatin or that due to the accumulation of proliferation genes the chromatin has adopted a more open chromatin environment.

### Analyzing Chromosome Haplotypes Enables the High-Resolution Chromatin Structure Mapping of Homologous Chromosomes

**Figure 2:**
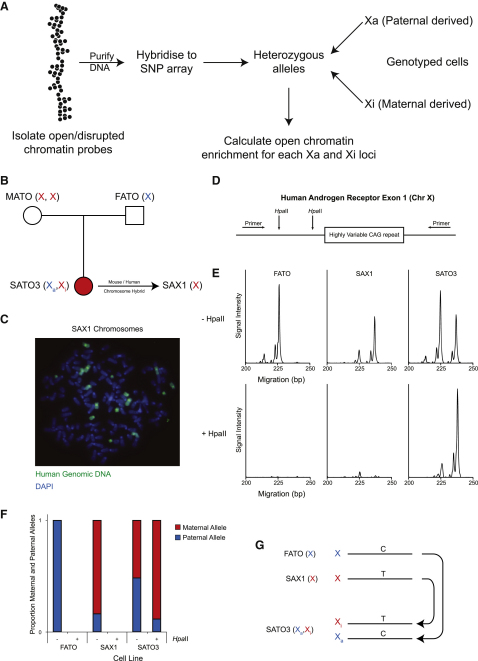
Mapping the Chromatin Fiber Structure of Homologous Chromosomes (A) Scheme for isolating open chromatin fibers from a female cell line and mapping their distribution across homologous chromosomes. (B) SATO3 cells were derived from a female individual and have two X chromosomes. A cell line prepared from SATO3's father (FATO) carries one X chromosome and the other X chromosome comes from the mother (MATO). SATO3's maternal-derived X chromosome has been isolated in a mouse/human chromosome hybrid (SAX1). See also Figure S2. (C) SAX1 cells hybridized to human genomic DNA (green). (D) To analyze the clonality of SATO3 cells with respect to X inactivation the two X chromosomes are distinguished by a highly variable CAG repeat at the androgen receptor locus. (E) PCR amplification of the CAG repeat from FATO, SAX1, and SATO3 cells and analysis of the product on a capillary sequencer distinguishes the two X chromosomes (the paternal X chromosome has a 225 bp PCR fragment while the maternal X chromosome has a 237 bp PCR fragment). PCR amplification in the presence of HpaII allows the transcriptionally inactive locus (DNA on the Xi chromosome is methylated) to be identified. (F) Quantification of the proportion of maternal and paternal alleles after HpaII digestion identifies SATO3 as a clonal cell line with respect to X inactivation. (G) The haplotype of SATO3 heterozygous SNPs are determined from FATO and SAX1 SNPs.

To investigate the relationship between chromatin fiber structure and transcription, we have used the X chromosome as a model. In female cells, the active (Xa) and the inactive X (Xi) chromosomes have almost identical DNA sequences so we can directly analyze the effect of transcription on chromatin structures. As described, we fractionated chromatin fibers and mapped them to the X chromosome by hybridization to genomic SNP arrays (Figure 2A). SATO3 female lymphoblastoid cells have two X chromosomes, one passed down from the father and the other from the mother (Figures 2B and 2C). The Xi in SATO3 cells has the typical appearance of forming a brightly staining Barr body that is enriched for H3K27me3 and macroH2A (Figures S2A–S2D) and genes (except XIST) are only expressed from the Xa chromosome (Figures S2E–S2H). To ensure the same X chromosome is inactive in each SATO3 cell, we amplified a variable CAG repeat on the X chromosome to distinguish between them. As many loci are methylated on the Xi compared with the Xa, the amplicon selected encompasses methylation sensitive restriction enzyme sites allowing us to discriminate between them (Figure 2D). This showed that SATO3 cells are clonal with the maternal-derived chromosome being silenced in each cell (Figures 2E and 2F). To determine the SNP haplotype of the active and inactive X chromosomes, we analyzed SNPs from FATO (paternal X chromosome) and SAX1 (maternal X chromosome) (Figure 2B) cells enabling us to determine the haplotype of 3965 heterozygous SNPs on the SATO3 X chromosomes (Figure 2G). We also genotyped the similarly sized chromosome 7 (HSA7), enabling us to separately examine its two homologs.

### Active and Inactive X Chromosomes Have Similar 30 nm Chromatin Fiber Structures

**Figure 3:**
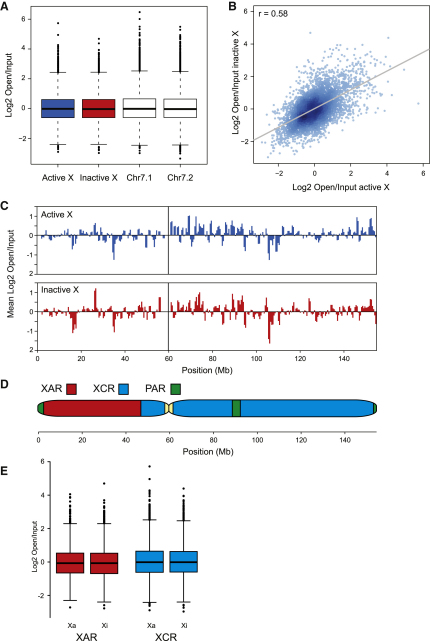
Active and Inactive X Chromosomes Have Similar Bulk 30 nm Chromatin Fiber Structures The open chromatin fraction was hybridized to Illumina SNP arrays versus an input chromatin control. The haplotype of heterozygous SNPs on chromosome X and HSA7 was used to distinguish between the chromatin structures of the two homologs. (A) Enrichment of open chromatin on homologous active and inactive X chromosomes. See also Figure S3. (B) Correlation between the enrichment of open chromatin on Xa and Xi chromosomes. (C) Distribution of open chromatin across Xa and Xi chromosomes in 1 Mb windows with a 500 kb step. (D) Schematic drawing of the X chromosome marking the evolutionary strata. X-added region (XAR), X-conserved region (XCR), pseudoautosomal region (PAR). (E) Enrichment of open chromatin at the XAR and XCR regions of the X chromosome.

It is unknown whether transcription-dependent changes in chromatin structure (Figures 1B–1D) are manifested through to the bulk 30 nm chromatin structure of chromosomes. The transcriptionally active X chromosome is packaged in euchromatin while the inactive X chromosome is packaged into facultative heterochromatin. Constitutive heterochromatin has a compact 30 nm chromatin fiber structure (Gilbert and Allan, 2001) so it is widely assumed that transcriptionally inactive facultative heterochromatin on the Xi will be organized in the same manner (Trojer and Reinberg, 2007). We therefore compared the chromatin structure of the Xa and the Xi chromosomes. Surprisingly, the Xa and the Xi are similarly enriched for open chromatin (Figure 3A), and the correlation between individual data points for them (Pearson's r = 0.58) (Figure 3B) are close to that observed for the chromosome 7 homologs (Figure S3) (Pearson's r = 0.55). Furthermore, examination of the patterns of open chromatin across the Xa and the Xi reveal highly related profiles (Figure 3C). The X chromosome can be divided into different parts based on its evolutionary organization: the X-conserved region (XCR) is 170 million years old while a more recent X-added region (XAR) was fused to the short arm of the ancestral X after X inactivation mechanisms had evolved (Kohn et al., 2004) (Figure 3D). Nearly all genes in the XCR are subject to transcriptional inactivation while the XAR region is highly variable with half the genes escaping inactivation (Carrel and Willard, 2005). Despite pronounced differences in transcription, similar chromatin structures are seen when we compare the XCR and XAR regions (Figure 3E). Therefore, transcription or a consequence of it is not sufficient to alter bulk 30 nm chromatin structure.

### Transcription Remodels 30 nm Chromatin Fiber Structures at Promoters

**Figure 4:**
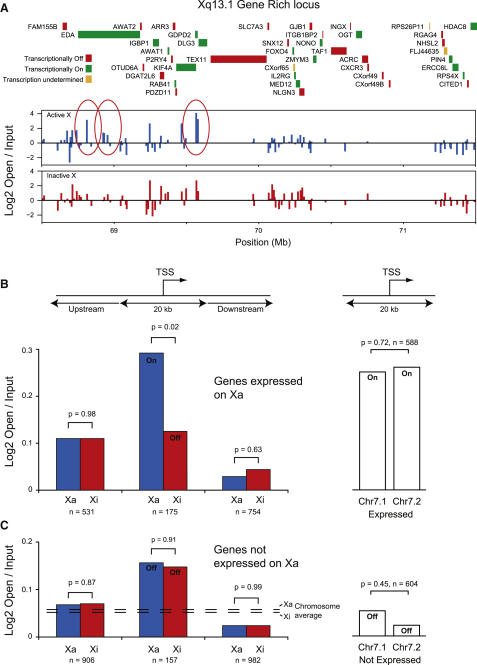
Transcription Remodels the Chromatin Structure of Promoters (A) Gene expression and high-resolution 30 nm fiber chromatin structure across the Xq13.1 gene-rich region in SATO3 cells. See also Figure S4. The structure of Xa and Xi were distinguished by characterizing the chromatin structure at heterozygous SNPs. Red ovals indicate regions showing a clear difference in enrichment between Xa and Xi. (B) Enrichment of open chromatin upstream, downstream and at the transcription start sites on the Xa and the Xi chromosomes for genes that are expressed on the X chromosome in SATO3 cells and for genes expressed on HSA7. (C) Enrichment of open chromatin upstream, downstream and at the transcription start sites on the Xa and the Xi chromosomes for genes that are not expressed on the X chromosome in SATO3 cells and for genes not expressed on HSA7. P values were calculated by a one-sided t test for paired samples.

Although chromosome-wide analysis indicated that the two X chromosomes had similar euchromatin-like 30 nm chromatin fiber structures (Figure 3A), our previous analysis also showed that transcriptionally active and inactive promoters were differently enriched in open chromatin (Figures 1B and 1F). We therefore hypothesized that the process of transcription might disrupt 30 nm chromatin fibers. To assess this, we identified gene-rich and gene-poor regions on the X chromosome at Xq13.1 and Xq25, respectively. We were unable to detect any gross differences in the chromatin fiber structures but we identified specific regions (marked as red ovals) in the enrichment of open chromatin in the vicinity of active genes on the Xa (Figures 4A and S4) but not the Xi consistent with transcription locally disrupting the 30 nm chromatin fiber.

To further examine this observation, we separated SATO3 X chromosome genes into expressed (Figure 4B) or not expressed groups (Figure 4C) and studied their chromatin structure. The chromatin structure at the TSS of equivalent X chromosome genes expressed in SATO3 cells (genes are expressed from the Xa, but not the Xi) (Figures S2E–S2H) show a pronounced difference in open chromatin (Figure 4B). Therefore, active genes on Xa have a more open promoter structure than the same genes found on the Xi. However, upstream and downstream regions have the same chromatin structure on the Xa and the Xi reinforcing our observation that the X chromosomes have the same 30 nm chromatin fiber structure away from promoters. Using a power calculation and effect size (details on request) based on the difference in structure between the Xa and the Xi promoters enabled us to estimate the sensitivity of our approach. The number of data points upstream and downstream of the promoter would therefore enable us see differences in chromatin structure of 0.08 log_2_ open/input units. This corresponds to a disruption smaller than the disruption found at transcriptionally inactive genes (Figure 4C).

The chromatin structure at the TSS of equivalent X chromosome genes not expressed in SATO3 cells is the same on the Xa and the Xi chromosome (Figure 4C). However, they do show an increase in open chromatin compared with the chromosome average or to upstream and downstream regions suggesting they are in a structurally poised state as found for other inactive genes (Figure 1B). As RNA polymerase and transcription factors are excluded from the Xi (Chaumeil et al., 2006), we can suggest that disruptions at inactive promoters on the Xi are formed by endogenous factors determined at the level of nucleosome interactions with the DNA (Cairns, 2009).

### Chromosome Territory Compaction Is Transcription Dependent

**Figure 5:**
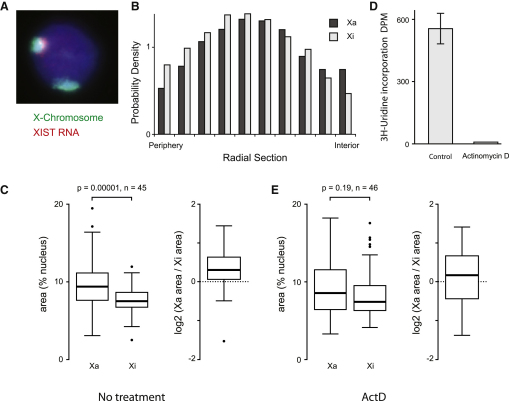
Chromosome Territory Compaction Distinguishes between Xa and Xi in a Transcription-Dependent Manner (A) Analysis of X chromosome territories by 3D DNA/RNA FISH in SATO3 female lymphoblastoid cells using DNA probes for the X-chromosome (green) and a probe against XIST (red). (B) Radial position of the two X chromosomes was determined by calculating the amount of territory signal present in images of nuclei that had been divided into rings of equal area (n = 89). (C) The area of the Xa and the Xi chromosome territories was measured from segmented nuclei (p values were determined by Wilcoxon test for paired samples). (D)Actinomycin D treatment inhibits transcription (error bar is ±SEM). (E) The area of the Xa and the Xi chromosome territories measured after transcription inhibition by actinomycin D (p values were determined by Wilcoxon test for paired samples). See also Figure S5.

The transcriptionally active euchromatic X chromosome and the heterochromatic inactive X chromosomes are cytologically very different (Rego et al., 2008). The cytological manifestation of the inactive X, the Barr body, is visible near to the transcriptionally repressive nuclear periphery in some cells depending on cell type and culture conditions. Other molecular markers of the Xi, like H3K27me3 or macroH2A, are also frequently visible in nuclei (Figures S2A–S2D). The perception is that the compact organization of the inactive X promotes or maintains its transcriptional inactivity and its location near to the nuclear periphery is thought to contribute to this (Zhang et al., 2007). To investigate whether the nuclear location of the Xi could influence its apparent compaction and transcriptional inactivity, we measured the radial position of the Xa and the Xi chromosomes in SATO3 cells using 3D RNA/DNA-FISH (Croft et al., 1999) (Figure 5A). The two X chromosomes occupy similar locations intermediate between the nuclear periphery and center (Figure 5B) showing that the nuclear location of the X chromosome is not sufficient to determine its compaction. Previously the transcriptionally active gene-rich HSA19 has also been shown to have a more decondensed organization than the gene-poor HSA18 (Croft et al., 1999) (Figure S5), but the compaction of the Xa and the Xi are still debated (Eils et al., 1996; Rego et al., 2008). Using 3D RNA/DNA-FISH, we measured how much of the nucleus was occupied by each X chromosomes and find the Xa occupies a significantly larger (median, 9.4%) area of the nucleus than the Xi (median, 7.5%), consistent with the Xi being more condensed (Figure 5C). This difference in size is similar to the difference between the HSA18 and the HSA19 territories but is slightly larger than found for another female cell line, RPE1, that also has a prominent Barr body (Figure S5). Inhibition of gene transcription in SATO3 cells (Figure 5D) promotes a condensation of the Xa chromosome territory to the level of the Xi territory (Figure 5E), showing that transcription inhibition is sufficient to promote territory compaction.

### Transcription-Dependent Large-Scale Chromatin Fiber Compaction

**Figure 6:**
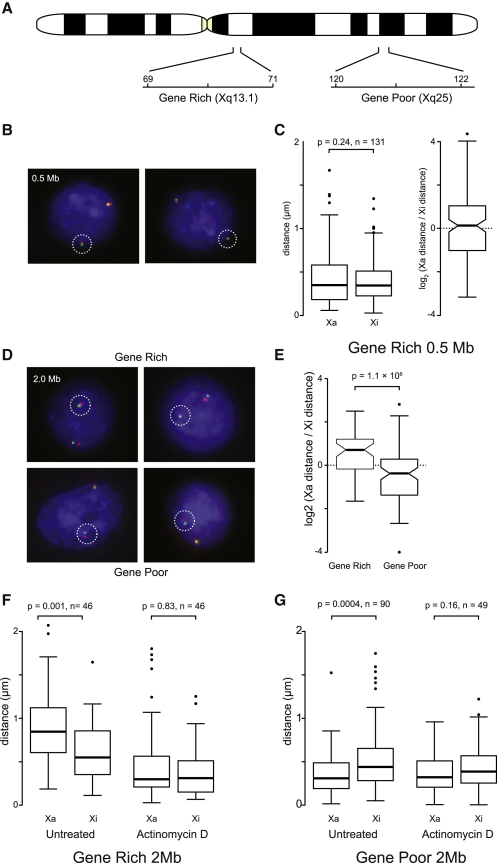
Large-Scale Chromatin Fiber Compaction Is Transcription Dependent (A) 3D DNA/RNA FISH was used to measure compaction of large-scale chromatin structures in SATO3 cells by using DNA probes separated by 0.5 or 2.0 Mb (pseudocolored in red and green) and a probe against XIST, in a gene-rich and -poor region. See also Figure S6. (B) The distance between probes 0.5 Mb apart measured from images of individual nuclei (the Xi is marked by a white circle). (C) Probe distances and ratio between the Xa and the Xi values were calculated for individual nuclei. (D) The distance between probes 2.0 Mb apart measured from images of individual nuclei (Xi is marked by a white circle). (E) Ratio between the Xa and the Xi values were calculated for individual nuclei. (F and G) Probe distances for the gene-rich and gene-poor regions before and after transcription inhibition. P values were calculated by Wilcoxon test for paired samples.

Our results suggest that the visual appearance of compact large-scale chromatin structures, for example, the Barr body (Figure 5C) might be a reflection of transcriptional inactivity. As transcription can decondense chromatin structures, we reasoned that transcription might therefore have the capacity to alter large-scale chromatin fiber structures (Tumbar et al., 1999; Muller et al., 2001; Tsukamoto et al., 2000). To investigate whether the compaction of the X chromosome territories also corresponded to the compaction of large-scale chromatin structures we selected probe pairs 0.5 or 2.0 Mb apart in gene-rich and gene-poor regions at Xq13.1 and Xq25, respectively (Figure 6A) and analyzed the compaction of loci by 3D RNA/DNA FISH. Labeled DNA probes were hybridized together with a probe to XIST RNA on SATO3 cells and the distance between the probes measured (Figures 6B and S6A). In SATO3 cells, we observed no difference in the compaction of probes 0.5 Mb apart between the Xa and the Xi indicating that at this level of organization the two chromosomes are similar (Figure 6C). However, there was a large difference in compaction between the two X chromosomes using probes 2 Mb apart where there is a significant 1.5-fold difference in compaction between the gene-rich Xa and Xi loci (p = 0.001, Wilcoxon test for paired samples; Figures 6D–6F). Differences in compaction were also seen for another gene-rich locus at Xq22.1 in SATO3 cells and similar differences were seen in RPE1 cells (Figures S6B–S6F). Differences in large-scale compaction were also observed for regions of gene-rich (transcriptionally active) and gene-poor (transcriptionally inactive) regions on human chromosome 11 (HSA11) (Gilbert et al., 2004). Transcription inhibition induced by actinomycin D treatment promoted a substantial compaction of the Xq13.1 locus on the Xa to the level of the Xi (Figure 6F). In contrast, the gene-poor region at Xq25 on the Xa is normally more compact than the Xi but transcription inhibition promotes the loci on the two homologous chromosomes to adopt similar degrees of compaction (Figure 6G). By using a deletion as a marker, we determined that such differences were not observed between two HSA11 homologs (Figures S6G–S6J). Our data therefore show that active and inactive chromosomes can have different large-scale chromatin structures but have equivalent 30 nm chromatin fiber structures (Figures 3A, 4B, and 4C).

## Discussion

In the bulk of the mammalian genome, the fundamental 30 nm chromatin fiber is heterogeneous (Gilbert et al., 2004), dynamic (Henikoff, 2008), and interspersed with allelic-specific discontinuities (Boyle et al., 2008; McDaniell et al., 2010) creating points of flexibility. High-resolution mapping has now enabled us to map these open chromatin disruptions to the transcription start sites of genes (Figure 1A). This assay for measuring chromatin fiber structure is sufficiently sensitive to enable us to detect small disruptions at the promoters of inactive genes (Figure 1B). These are probably reflective of a nucleosome free region or small disruptions at transcription factor binding sites, and these disruptions are enhanced by the process of transcription (Figures 1C, 1D, and 4B). There is a linear relationship between chromatin fiber sedimentation and log_2_ (open/input) enrichment of chromatin that can be related to the sedimentation properties of differently shaped chromatin fibers (Figures S1H–S1M). Modeling of our sedimentation data enabled us to estimate possible chromatin fiber structures around the TSS of genes (Figure 1D). Gene-rich chromosomes are enriched in disrupted chromatin that is additive creating open chromatin domains (Figure 1E). As we now show that the transcriptionally active X chromosome has the same bulk chromatin structure as the inactive X chromosome (Figure 3), we can also suggest that the difference in 30 nm chromatin structure between gene-rich and gene-poor chromosomes is not caused by a fundamental transcription-dependent difference in the bulk 30 nm chromatin fiber. Surprisingly different classes of genes have very different chromatin fiber structures (Figure 1E). Do transcriptionally active genes create open chromatin domains or have genes been preferentially located in regions of the genome that have an open structure? Transcription clearly has the capacity to open chromatin (Figure 4B) presumably by the transcription and associated machinery disrupting the canonical chromatin fiber (Shivaswamy et al., 2008). However, at transcriptionally inactive genes where transcription cannot be directly influencing the chromatin, there is a pronounced difference in chromatin fiber structures between gene classes (Figure 2E) clearly indicating that different genes are preferentially located in specific chromatin environments. Our data show that proliferation (housekeeping) genes are associated with an open chromatin environment compared with tissue-restricted genes (Lercher et al., 2002; Caron et al., 2001; Versteeg et al., 2003). This suggests that genes located in an open chromatin environment are more readily expressed and would be associated with increased transcriptional noise. In contrast restricted genes are in a closed chromatin environment protecting them from aberrant activation. This could have important implications for diseases like cancer where genes located in an open chromatin environment are more likely to become misexpressed compared with genes in a closed chromatin environment (Versteeg et al., 2003). Likewise, if due to changes in genomic organization closed chromatin regions were to become opened, then tightly regulated genes are more likely to be activated, causing the aberrant expression of potentially damaging transcriptional regulators.

Gene-rich chromosomes are enriched in open chromatin a considerable distance from transcription start sites (Figures 1E, S1A, and S1B). Gene-rich chromosome territories have been shown to intermingle more than gene-poor chromosomes (Branco and Pombo, 2006) and recent genome-wide studies (Lieberman-Aiden et al., 2009) have shown that open chromatin regions interact with other open regions. Gene-rich regions cluster in SC-35 domains that are enriched in metabolic factors (Shopland et al., 2003) and in doing so the intrachromosomal interactions could promote the formation of chromatin disruptions at the level of the 30 nm fiber establishing open chromatin domains (Schoenfelder et al., 2010). This would suggest that large-scale levels of chromatin folding could then influence the packaging of the 30 nm chromatin fiber and modulate transcriptional potential. However, recent studies showing allelic-specific differences in DNaseI hypersensitive sites and transcription factor binding (McDaniell et al., 2010; Kasowski et al., 2010) and our data showing differences in structure between active and inactive promoters (Figures 1B, 4B, and 4C) suggest that localized effects on the chromatin fiber are not manifest through to the bulk 30 nm chromatin fiber (Figures 3C, 4B, and 4C). Likewise, although we demonstrate that the process of transcription influences large-scale chromatin structures (Figure 6), it is unlikely that local disruptions at TSS are sufficient as they do not affect the bulk chromatin fiber.

In human female cells, one of the X chromosomes is inactivated and packaged into cytologically distinct facultative heterochromatin, where it shows a permanent positive heteropycnosis and is transcriptionally inactive (Rego et al., 2008). In other species, such as locusts, in male germ cells the X chromosome switches between negative and positive heteropycnosis during development (Nur, 1978) showing that the formation of facultative heterochromatin can be reversible. In differentiating erythrocytes, facultative heterochromatin formation correlates with the expression of the variant linker histone H5 (Gilbert et al., 2003) causing a reduction in gene transcription and cell replication and concomitantly the nucleus becomes heteropycnotic. Cytologically facultative heterochromatin is the visual appearance of condensed chromatin, but at a molecular level it also has many of the hallmarks of transcriptionally inactive chromatin. In our study, we have shown that despite gross differences in transcription the Xa and the Xi have similar bulk chromatin fiber structures (Figure 3A). This is in contrast to the clear differences between the two chromosomes at a large scale of chromatin folding. The two chromosomes are cytologically different with the Xi forming a Barr body, the Xi territory is compact compared with Xa and is composed of more compact large-scale chromatin fibers. Furthermore, transcription inhibition promotes a compaction of large-scale chromatin structures on the Xa to a level seen for the Xi (Figure 6D). Therefore, the cytological appearance of facultative heterochromatin is potentially due to its transcriptional inactivity. In contrast, constitutive heterochromatin is associated with repetitive DNA that positions nucleosomes in a regular manner. This regular organization enables the formation of a stable canonical 30 nm chromatin fiber that is tightly packaged (Gilbert and Allan, 2001). However, the cytological appearance of constitutive heterochromatin may also be influenced by its transcriptional state. In HeLa cells heat shock induces satellite 3 (sat3) transcription from HSA9 (Jolly et al., 2004) causing a decompaction of the sat3 locus. Blocks of sat3 are occasionally visible by DAPI staining in normal cells but these are rarely visible after heat shock (data not shown).

Our data show that the process of transcription promotes the decondensation of large-scale chromatin structures independent of the underlying 30 nm chromatin fiber. This observation seems surprising as large-scale chromatin structures in cells are formed from the folding of 30 nm chromatin fibers. Gene-rich, transcriptionally active, regions are associated with many chromosomal interactions (Lieberman-Aiden et al., 2009), and our data suggest that this might influence the folding of the 30 nm fiber (Figure 2D). Interestingly, a component of the PRC1 complex, RING1B, has been shown to alter large-scale chromatin structures, possibly by repressing transcription (Eskeland et al., 2010). The process of transcription might therefore disrupt intrachromosomal interactions causing a decompaction of chromatin fibers. However, 3C and 4C techniques have shown that although transcription inhibition reduced RNA polymerase binding to regulatory elements intramolecular interactions are unaffected (Palstra et al., 2008). Genome-wide the DNA packaged into chromatin is in a relaxed conformation (Sinden et al., 1980) but at transcriptionally active genes the DNA is in a negatively supercoiled configuration (Giaever and Wang, 1988), and this is believed to enhance transcription efficiency (Dunaway and Ostrander, 1993). The process of transcription introduces positive supercoils in front of polymerase while negative supercoils are introduced behind the polymerase (Liu and Wang, 1987). Supercoils are released by the partial uncoiling of nucleosomal DNA, and it has been shown that histone acetylation also has the ability to release negative supercoils (Norton et al., 1989). Some supercoils are absorbed by the chromatin fiber while others are removed by topoisomerases (Stewart et al., 1990) that are part of the polymerase complex. DNaseI sensitivity of the active β-globin locus is rapidly lost following topoisomerase II inhibition by novobiocin (Villeponteau et al., 1984) and treatment with novobiocin can also block the *Drosophila* Hsp70-induced heat shock response (Han et al., 1985) implying that supercoiling can disrupt chromatin structure. Positive supercoils accumulating in genes can be dissipated over about 100 kb (Joshi et al., 2010) suggesting that supercoiling in active regions could be propagated through the chromatin fiber to influence the folding of large-scale chromatin structures.

Our high-resolution chromatin structure map of active and inactive regions of the human genome has enabled us to show that transcription promotes the formation of open chromatin around promoters influencing higher order levels of chromatin folding independently of the 30 nm chromatin fiber. We also suggest that the appearance of facultative heterochromatin is a visual manifestation of large-scale chromatin structures, formed from euchromatin, in response to transcriptional inactivity. It will now be interesting to determine how transcriptional processes at the level of the 30 nm fiber can directly affect higher levels of chromatin organization.

## Experimental Procedures

### Cell Culture, Chromatin Fractionation, and Microarray Hybridization

SATO3 and FATO lymphoblastoid cells were cultured as described previously (Gilbert et al., 2004). To inhibit transcription, cells were treated with 50 μg/ml actinomycin D for 3 hr. Chromatin was prepared and hybridized to microarrays as described previously (Gilbert et al., 2004) with modifications (Supplemental Experimental Procedures).

### Gene Expression Data

RNA was prepared from cells in triplicate using Tri-Reagent (Sigma) extraction and isopropanol precipitation. RNA was hybridized to expression arrays (Supplemental Experimental Procedures).

### 3D RNA/DNA Four-Color FISH and Image Capture and Analysis

Nonadherent lymphoblastoid cells (2 × 10^4^ cells) were cytospun on to glass slides at 600 rpm for 10 min and processed using standard techniques with minor modifications (Supplemental Experimental Procedures). Slides were imaged using a Zeiss Axioplan II fluorescence microscope with Plan-neofluar objectives and images were processed using custom IPLab scripts (Supplemental Experimental Procedures).

### Mapping Chromosome Haplotypes and SNP Array Data Processing

The method used to determine chromosome haplotypes is described in detail in the Supplemental Experimental Procedures. All cell lines were genotyped by hybridizing to Illumina Human HapMap 550K Genotyping SNP Arrays. SATO3 and FATO cells were genotyped using standard clustering of SNPs from Illumina's Bead Studio (Version 3.13) software. SNP Array Data were processed using the R statistical software and the limma Bioconductor Package.

## Accession Numbers

Expression, genotyping, and chromatin structure data have been deposited in the Gene Expression Omnibus under accession code GSE23818.

## Supplemental Information

Document S1. Supplemental Experimental Procedures, Supplemental References, and Six Figures
