# Supplementary material for: Analysis of Active and Inactive X Chromosome Architecture Reveals the Independent Organization of 30 nm and Large-Scale Chromatin Structures
Source: Mol Cell. 2010 Nov 12;40(3):397–409. doi: 10.1016/j.molcel.2010.10.013 (PMC3038259; doi:10.1016/j.molcel.2010.10.013)
Supplement: Document S1. Supplemental Experimental Procedures, Supplemental References, and Six Figures [file mmc1.pdf]

Supplemental Information

Molecular Cell, *Volume 40*

Analysis of Active and Inactive X Chromosome Architecture Reveals the Independent Organization of 30 nm and Large-Scale Chromatin Structures

Catherine Naughton, Duncan Sproul, Charlotte Hamilton, and Nick Gilbert

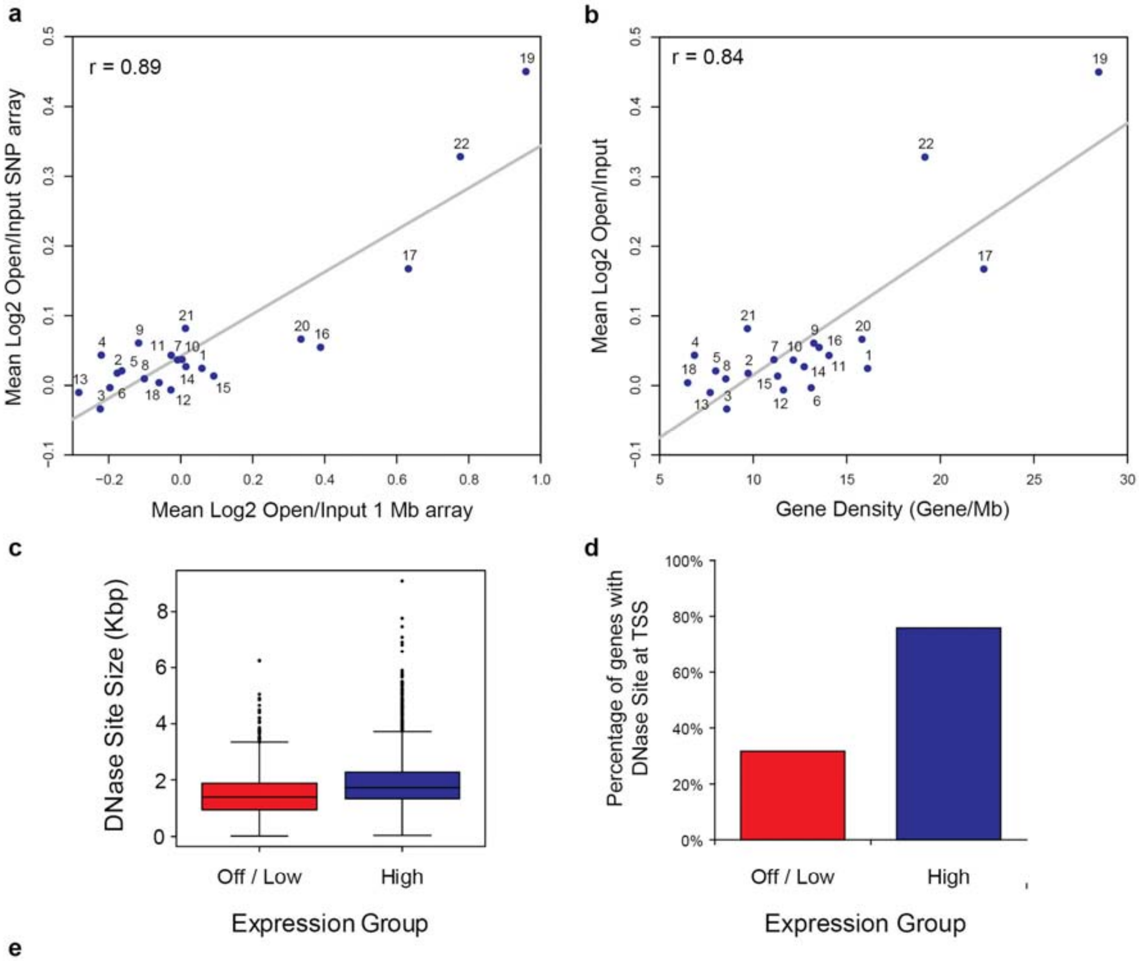

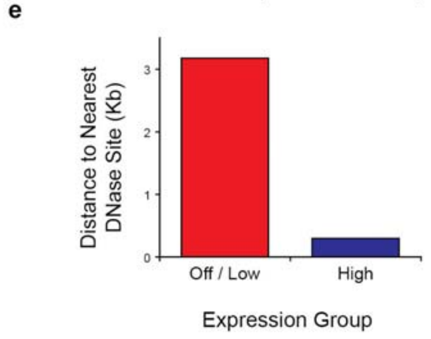

f

	% genes	
	Chr 18	Chr 19
Proliferation	61.5	65.7
Early Differentiation	15.9	6.3
Lineage Specification	22.5	28.0

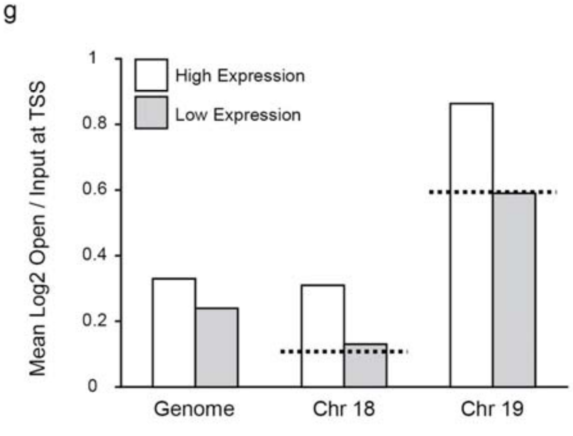

Naughton et al., Supplementary Figure 1

h

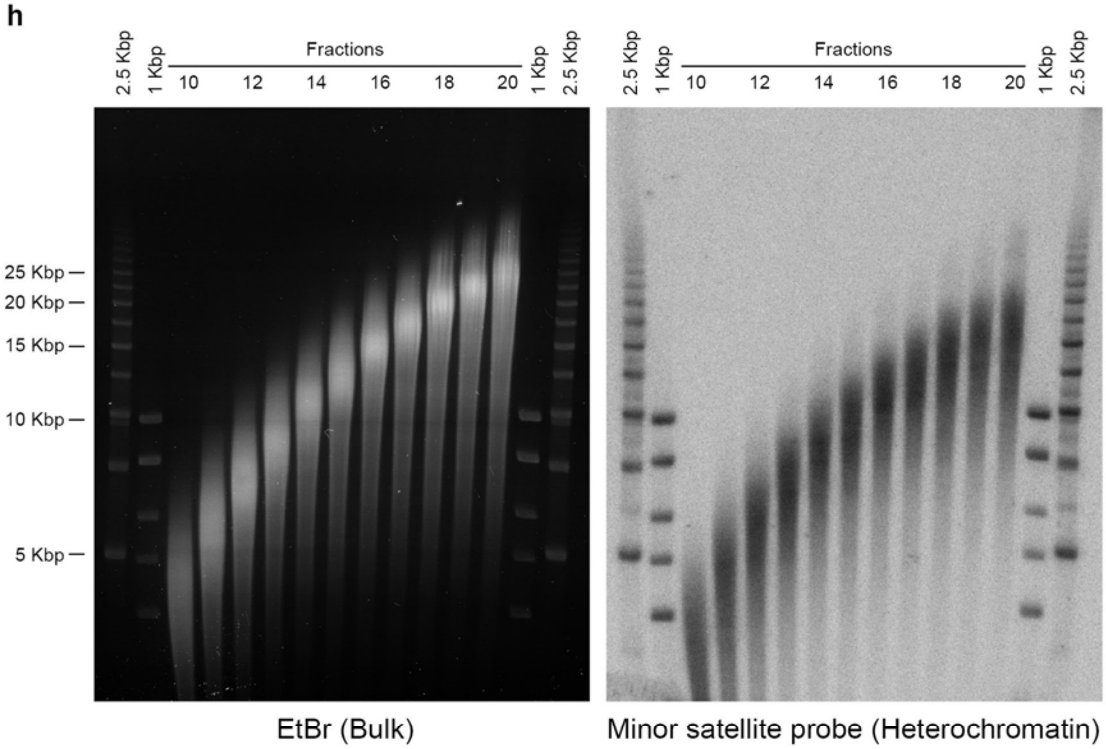

i

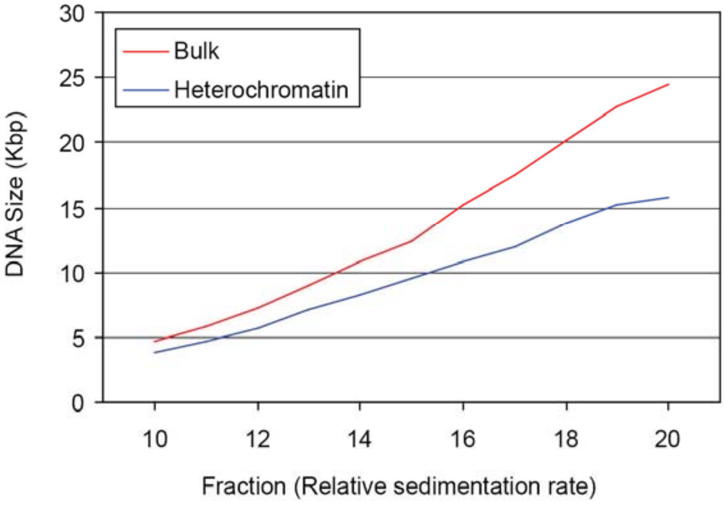

Naughton et al., Suppleleemntary Figure 1

j

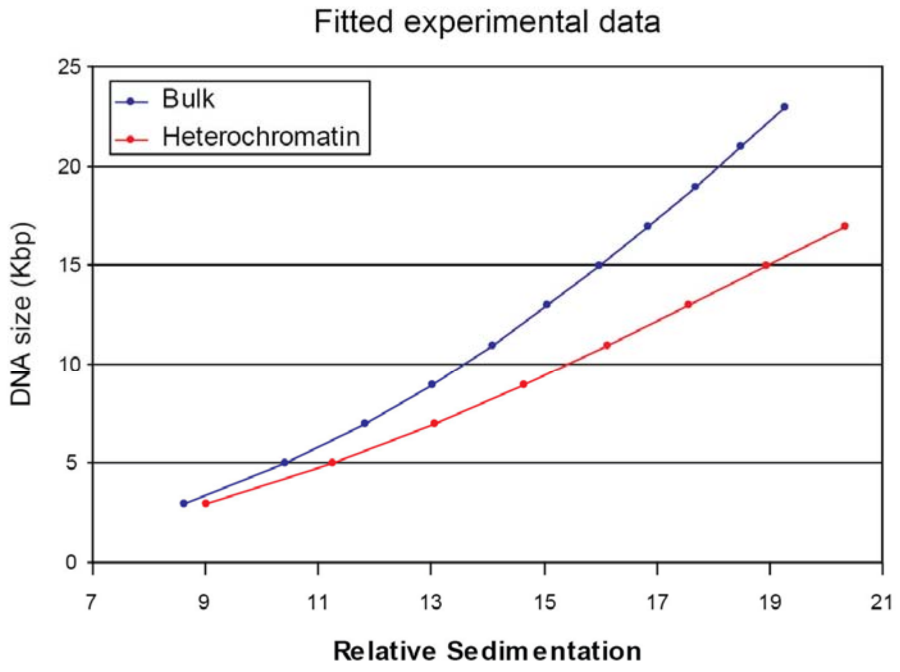

k

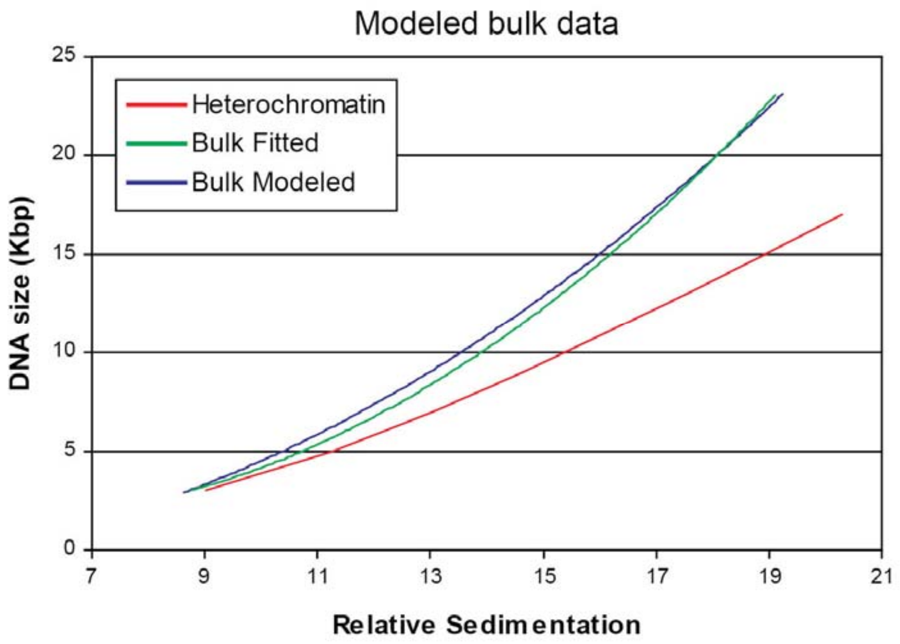

Naughton et al., Suplelemntary Figure 1

l

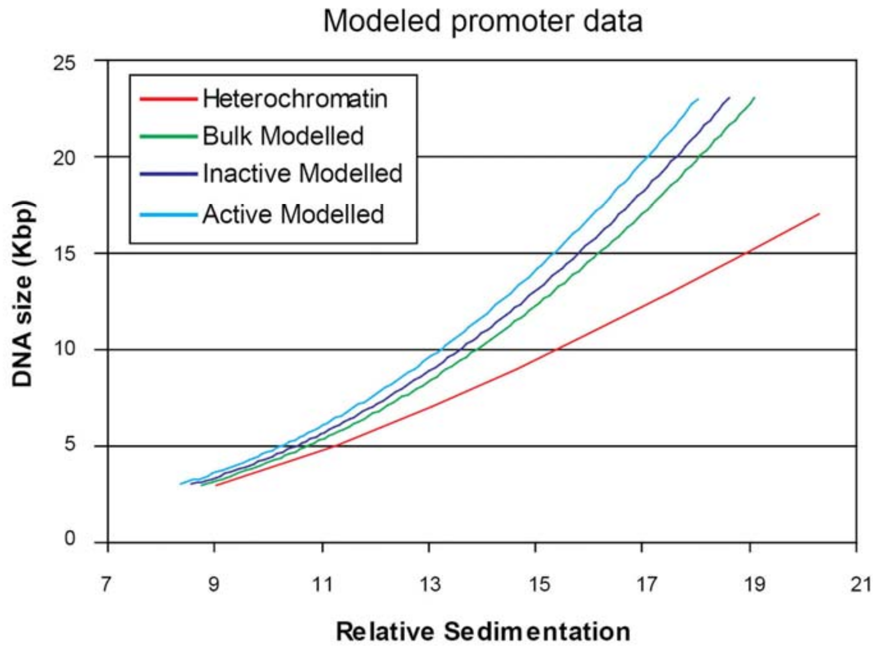

m

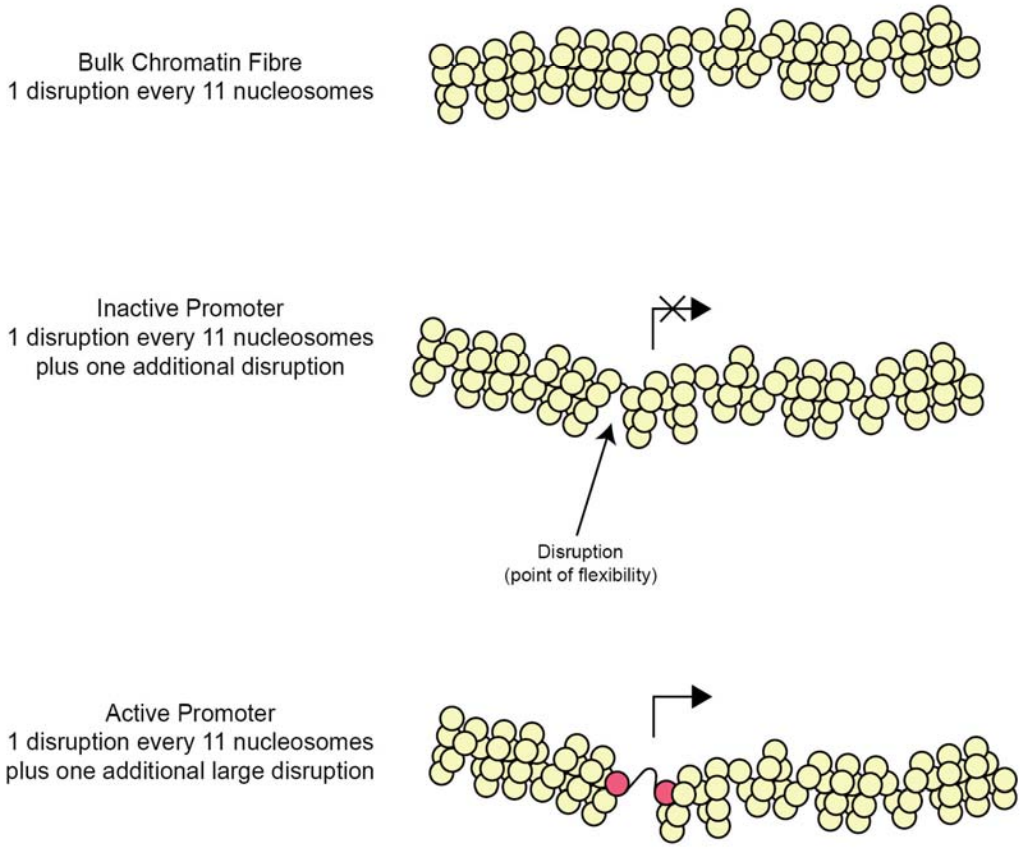

Naughton et al., Supplelemntary Figure 1

Figure S1. Distribution and Analysis of Disrupted Chromatin across the Human Genome Using Illumina SNP Arrays

This figure relates to figure 1. **(a)**. To ensure Illumina SNP arrays performed similarly to BAC arrays (Gilbert et al., 2004) we correlated the amount of disrupted chromatin on each chromosome from the SNP arrays to a data set obtained from BAC arrays (Pearson's r = 0.89). **(b)**. Correlation between the enrichment of "open" chromatin on each chromosome and gene density (Pearson's r = 0.86). Individual chromosomes are numbered. **(c)** High resolution mapping has been undertaken of DNaseI sites in CD4+ T-cells (Boyle et al., 2008). Re-analysis of their data shows that both transcriptionally active and inactive genes are associated with DNaseI sensitive sites that are approximately 1.6 and 1.3 Kbp in size, respectively. **(d)**. The frequency of a DNaseI site at a TSS is increased for expressed genes consistent with the increased likelihood of a disrupted chromatin structure (Figure 1c). **(e)**. Highly expressed genes are located near to other DNaseI sensitive sites consistent with the spreading of disrupted chromatin to adjacent regions (Figure 1e). **(f)**. Classes of genes found on gene poor HSA18 and gene rich HSA19. **(g)**. Proliferation genes are found in more open chromatin environments on HSA18 and HSA19 compared to the average chromatin structure of all TSS on the chromosome (dotted line).

Experimental modelling of 30-nm chromatin fibre organisation around transcription start sites.

Although it is impossible to determine the exact structures adopted by different chromatin fibres we have developed our modelling approach (Gilbert and Allan, 2001) on our data to predict possible chromatin structures found at the promoters of active and inactive genes.

All raw data and full calculations are available on request

For the idealised, canonical higher-order 30-nm chromatin fibre, the sedimentation coefficients of fibres with the same nucleosomal repeat length will be a function of their molecular weight (M), fibre lengths (l) and frictional ratios (f/f_0) as given by the following equation (Harrison and Klug, 1966)

$$s = k \frac{\sqrt[3]{Ml}}{(f/f_0)} \qquad (1)$$

Our analyses provide experimental data (Figure S1h, i and (Gilbert and Allan, 2001)) for the sedimentation of bulk-derived and satellite containing NIH3T3 mouse chromatin fibres, enabling us to derive a relationship between fibre length and frictional ratio according to equation 1.

Figure S1h. Soluble chromatin isolated from mouse cells was fractionated in an isokinetic sucrose gradient and the DNAs purified from individual fractions were sized on a pulsed field gel. After blotting to a membrane the samples were probed for mouse minor satellite (which we have previously shown has a canonical chromatin structure). **Figure S1i**. The size of the DNA contained in each gradient fraction was determined from densitometer traces of lanes from the ethidium bromide stained gel and the phosphorimages of hybridised filters. The relationship between DNA size and sedimentation velocity (fraction number) reveals the difference in conformation between satellite and bulk chromatin.

Our previous studies (Gilbert and Allan, 2001) indicated that the relationship between curves relating DNA size (fibre length) and sedimentation velocity (Figure S1i) for satellite-containing heterochromatin and bulk chromatin shows that satellite-containing chromatin adopts a more regular helical structure than bulk chromatin. By using equation 1, we calculated the relative sedimentation

coefficients (s/k) for a range of lengths of a regular 30-nm diameter chromatin fibre comprising 6 nucleosomes per helical turn, a pitch of 11-nm, a repeat length of 190 bp and the frictional ratio derived from experimental data (**Figure S1j**). To model the effect of higher-order fibre irregularity on fitted data we assumed that in bulk chromatin there is a point of disruption that increases the frictional coefficient and therefore reduces the sedimentation coefficient of a chromatin fibre and that the probability of such a disruption is a function of fibre length as described by equation 2.

$$Sd = Su - \left(\Pr \times l \cdot Ed \times Su\right) \quad (2)$$

Su = sedimentation of an undisrupted fibre

Sd = sedimentation of a disrupted fibre

Pr = probability of a disruption

Ed = extent disruption

l = length fibre

Data was generated using this approach and the relationship between the curves obtained for disrupted (bulk) fibres bears a close resemblance to fitted experimental data (**Figure S1k**).

Our array data showed that transcription start sites are associated with an increased probability of a disruption (Figure 1b). To model the structure of an inactive promoter we assumed the promoter had an extra disruption of the same magnitude of a normal disruption (equation 3) whilst for an active promoter we assumed the extra disruption had an increased magnitude (Df) (equation 4), possibly seen as a DNaseI sensitive site formed by the loss of a weakly associated nucleosome (Schones et al., 2008; Jin et al., 2009).

$$S_{inactive} = Su - \left(\left(\Pr \times l \cdot Ed \times Su\right) + \left(Ed \times Su\right)\right) \quad (3)$$

$$S_{active} = Su - ((\Pr \times l \cdot Ed \times Su) + (Ed \times Df \times Su)) \quad (4)$$

Df = disruption factor

To fit the theoretical model to experimental data we reasoned that the log2 Open/Input ratio is linearly related to the relative sedimentation rate because the chromatin was fractionated on an isokinetic sucrose gradient giving a linear relationship between sedimentation rate and extent of sedimentation. Therefore, as our “open” chromatin probes were derived from chromatin fragments that had 19 Kbp DNA we could fit an experimental estimate of sedimentation (from Figure 1a) to the modelled structure. By estimating the position of canonical heterochromatin in the log2 Open/Input ratio (Figure 3c and data from other chromosomes) and by taking the position of bulk chromatin as the genome average we can fit the modelled data to the experimental data for a 19 Kbp chromatin fragment (**Figure S1l**). Fitting the experimental data to the model for active promoters then gives a disruption factor (Df) of 2.25

Figure S1m. From this we conclude the bulk fibre has one disruption every 11 nucleosomes and an inactive promoter has one disruption every 11 nucleosomes plus one extra disruption. In contrast an active promoter has one disruption every 11 nucleosomes plus one additional large disruption, possibly caused by the loss of a nucleosome. These sites of disruption introduce new points of flexibility to the chromatin fibre and will form accessible points for the association of transacting factors. The disruption at active genes could then be stabilised by H2A.Z containing nucleosomes (Hartley and Madhani, 2009) (Figure S1m, red balls).

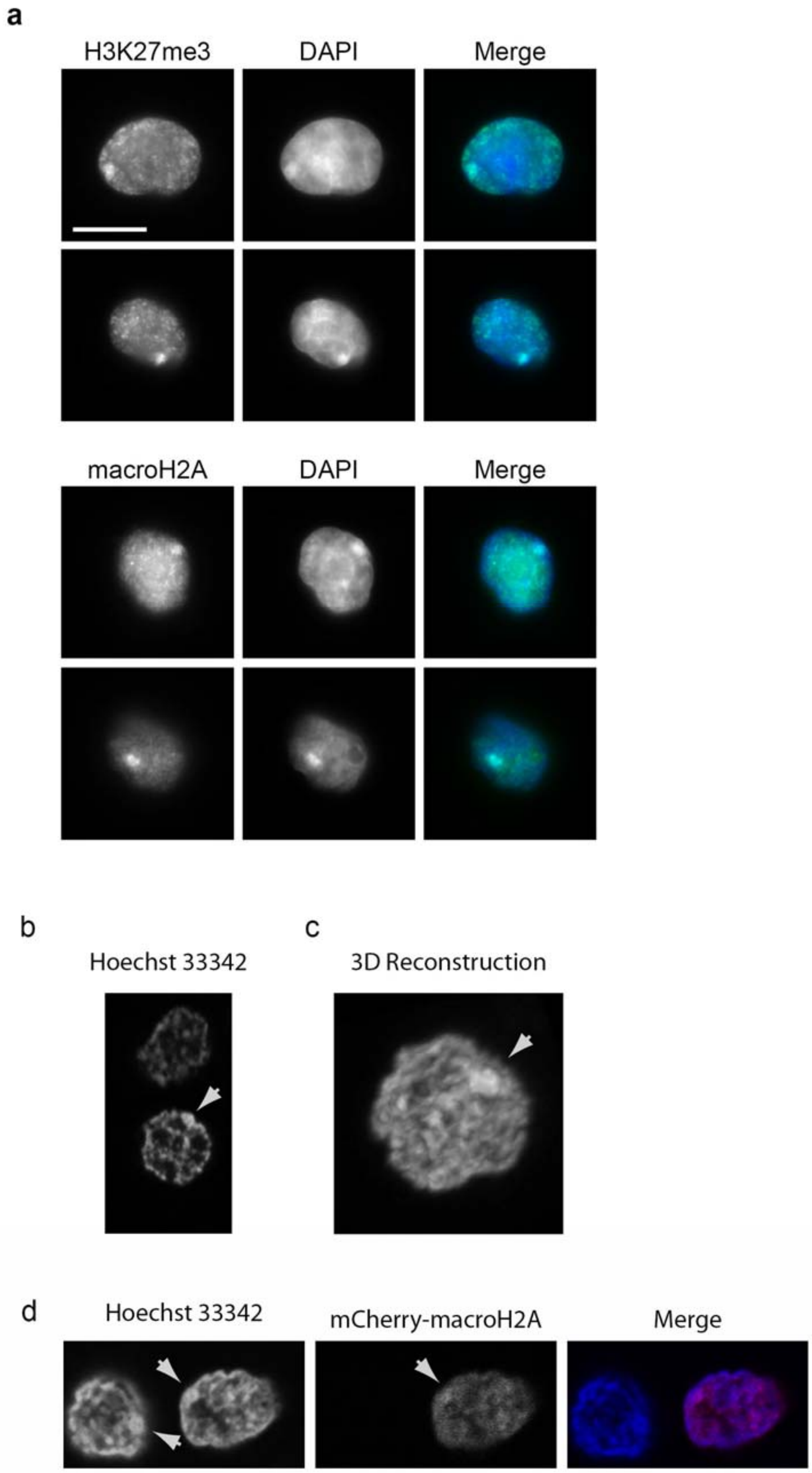

Naughton et al., Supplementary Figure 2

e

Gene	SNP	Allele	Maternal	Paternal	Position	Location
ATRX	rs3088074	C/G	C	G	76744108	coding
ATP7A	rs2227291	C/G	G	C	77074647	coding
SH3BGRL	rs1044828	C/T	T	C	80359374	3utr
GPC4	rs1048369	C/T	T	C	132162857	coding
CD99L2	rs6877	A/G	A	G	149605668	3utr
XIST	rs1620574	C/T	C	T	72826838	XIST

f

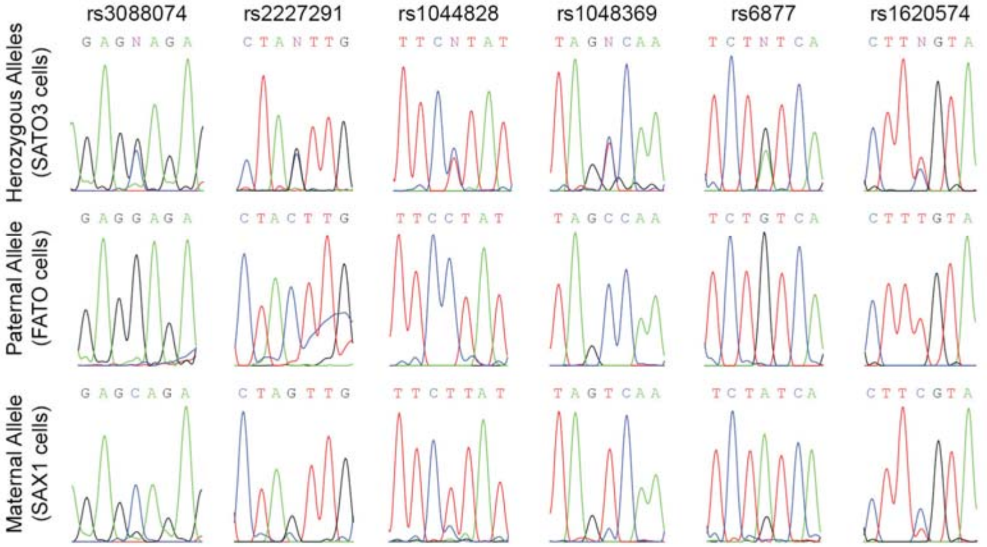

g

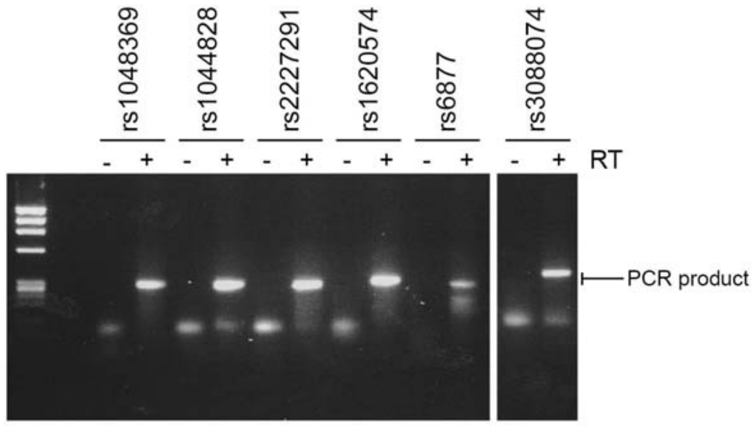

h

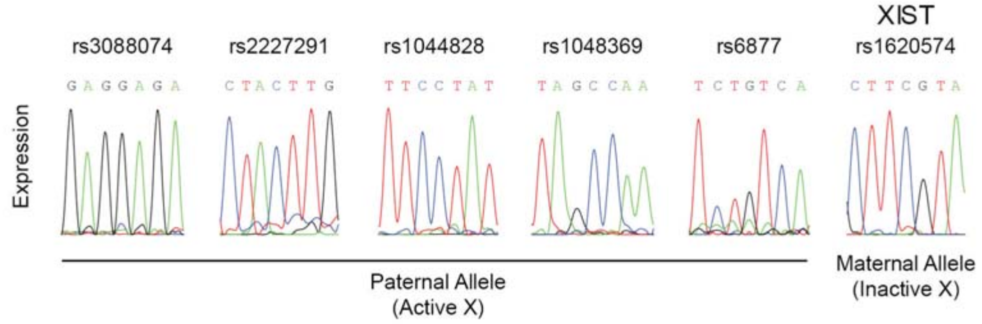

Naughton et al., Supplementary Figure 2

Figure S2. Characterising the X chromosomes of SATO3 cells

This analysis relates to figure 2. **(a)** Barr body identification in fixed SATO3 cells and staining for H3K27me3 and macroH2A, known to be enriched on the Xi. Scale bar = 10 μ. **(b)** Live cell imaging of Barr bodies in SATO3 cells using a confocal microscope. Cells were embedded in an agarose matrix, stained with Hoechst 33342. **(c)** 3D reconstruction of stacks through a nucleus showing the Barr body. **(d)**. Transfection and imaging of SATO3 cells transfected with a plasmid expressing mCherry-macroH2A. In transfected cells the Barr body (arrow) is enriched for macroH2A. **(e)**. Genes on the X chromosome are differentially expressed from the Xa and the Xi. Selected genes on HSA X that have coding heterozygous SNPs. **(f)**. Sanger sequencing to determine the haplotype of each allele on the two X chromosomes. **(g)**. Reverse transcription of RNA from SATO3 cells to produce cDNA followed by PCR amplification. **(h)**. Sanger sequencing of PCR amplified cDNA to identify which allele is expressed.

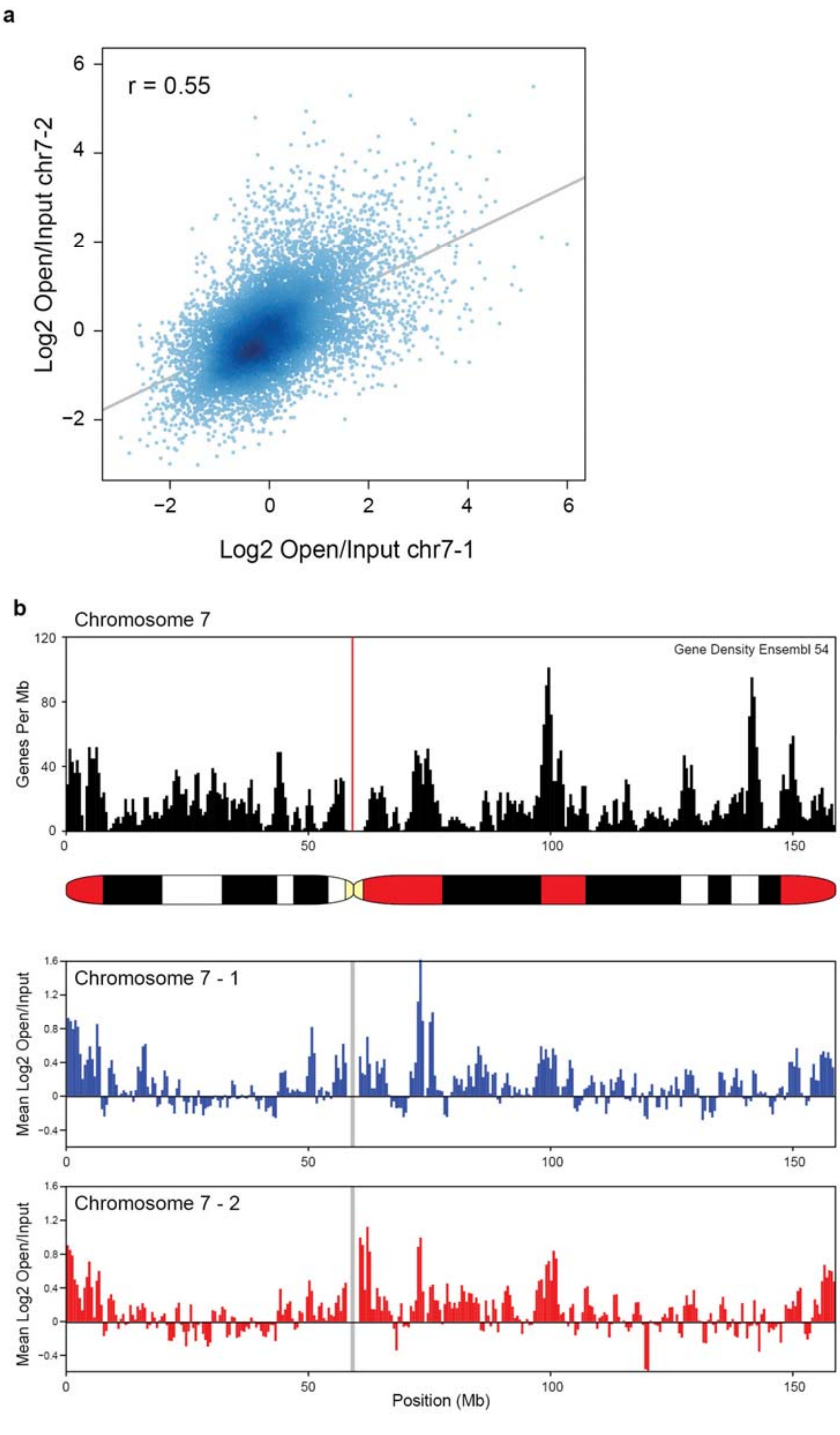

Naughton et al., Supplementary Figure 3

Figure S3. Distribution of disrupted chromatin across human chromosome 7 homologues

This analysis relates to figure 3. **(a)**. Correlation between the enrichment of disrupted chromatin at each heterozygous SNP for homologous Chromosome 7s (Pearson's $r = 0.55$). **(b)**. Enrichment of disrupted chromatin on each homologous chromosome 7s (data points are means over a 1 Mb window with a 0.5 Mb step).

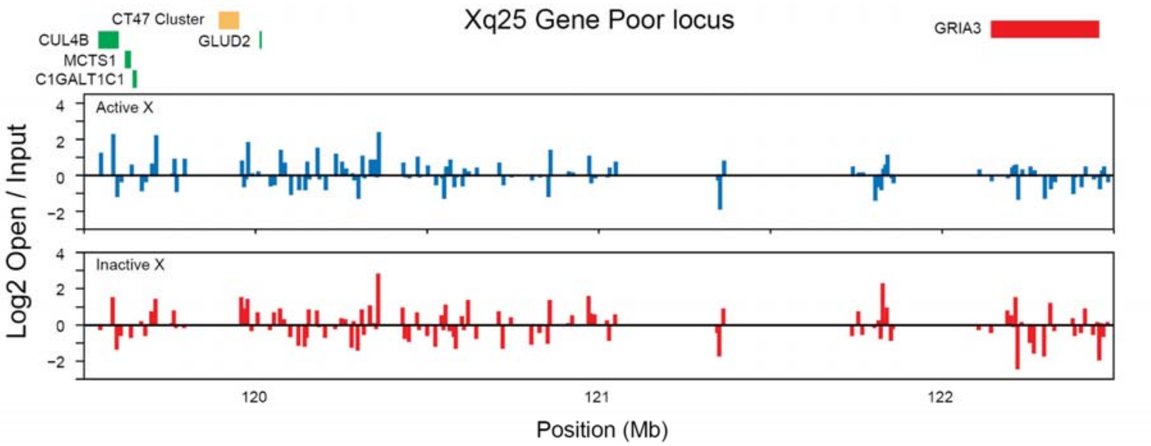

Naughton et al., Supplementary Figure 4

Figure S4. Distribution of open chromatin at a gene poor locus at Xq25

This data relates to figure 4. Gene expression and high resolution 30-nm fibre chromatin structure across the Xq25 gene poor region in SATO3 cells. The structure of the two X chromosomes were distinguished by characterising the chromatin structure at heterozygous SNPs.

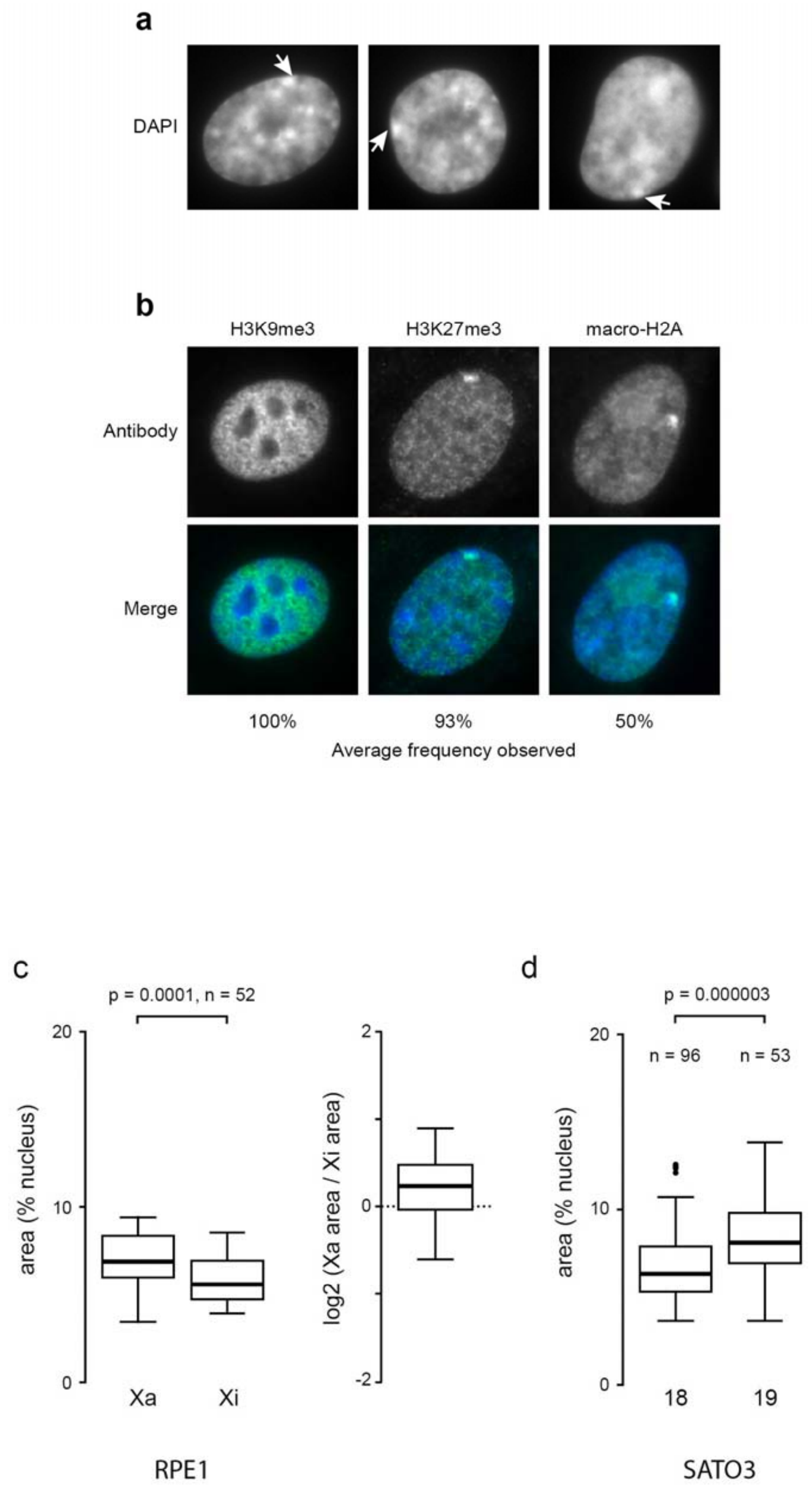

Naughton et al., Supplementary Figure 5

Figure S5. Cytological and molecular marks of the Xi and four colour RNA/DNA FISH to measure X chromosome compaction

This data relates to figure 5. **(a)**. The Barr body in RPE1 cells is visible in approximately 10% of nuclei by DAPI staining (marked by white arrow). **(b)**. In contrast molecular marks of the Xi like H3K27me3 and macro-H2A are visible in a higher proportion of nuclei. **(c)**. Analysis of X chromosome territories by 3D DNA/RNA FISH in RPE1 female cells using DNA probes for the X-chromosome and a probe against XIST. **(d)**. Analysis of HSA18 and HSA19 territories by 3D DNA FISH in SATO3 cells on separate slides using DNA probes against the territories labelled with the same fluorophores.

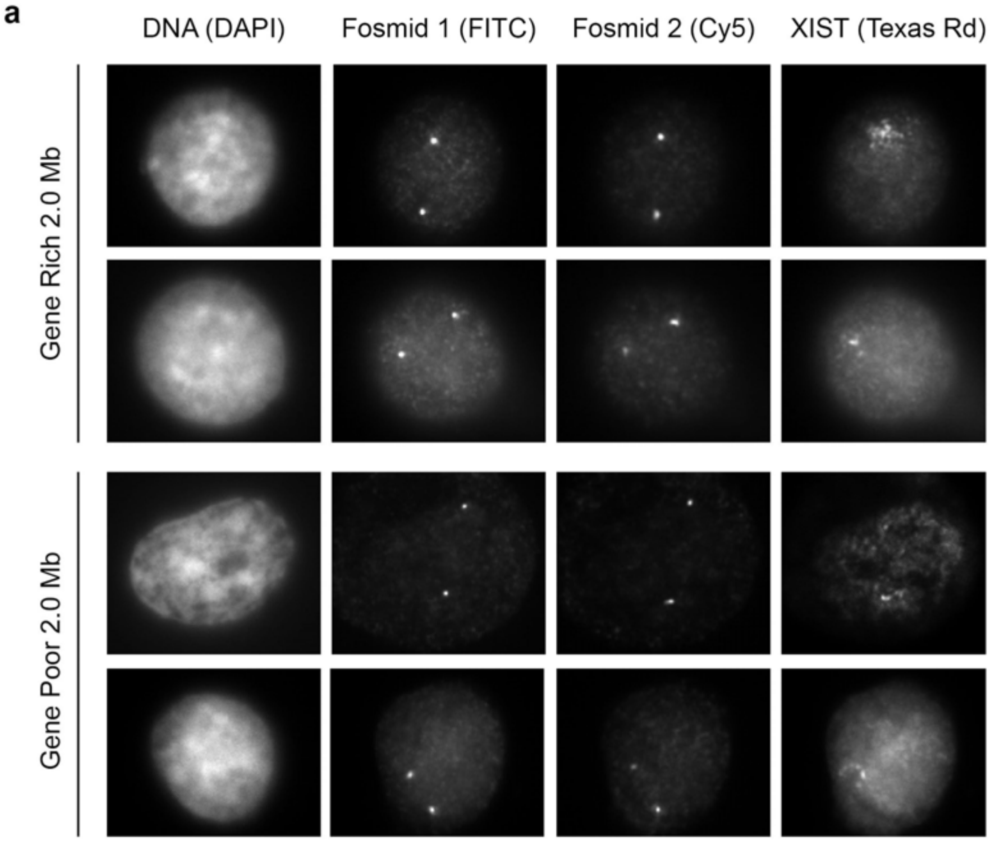

Naughton et al., Supplementary Figure 6

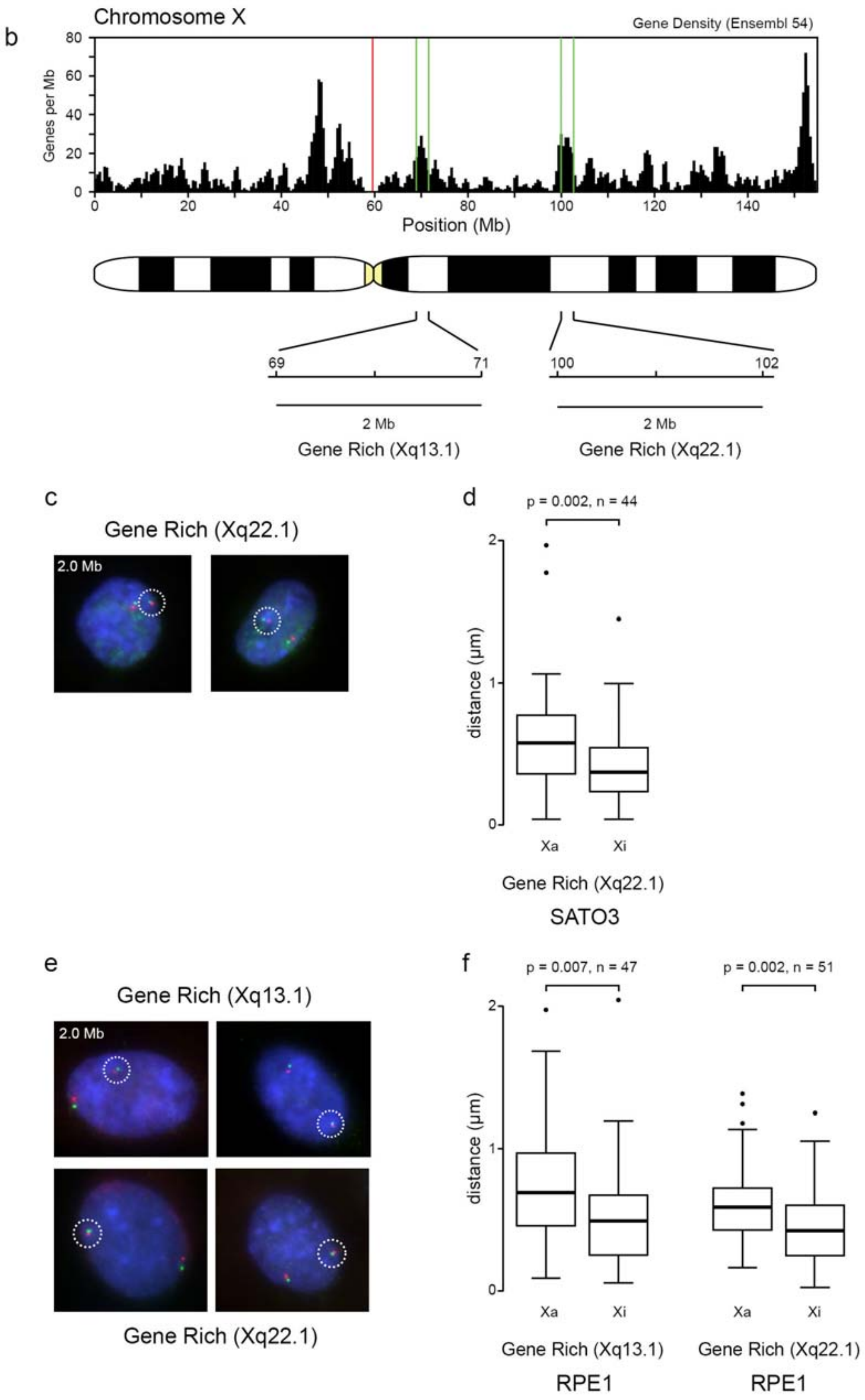

Naughton et al., Supplementary Figure 6

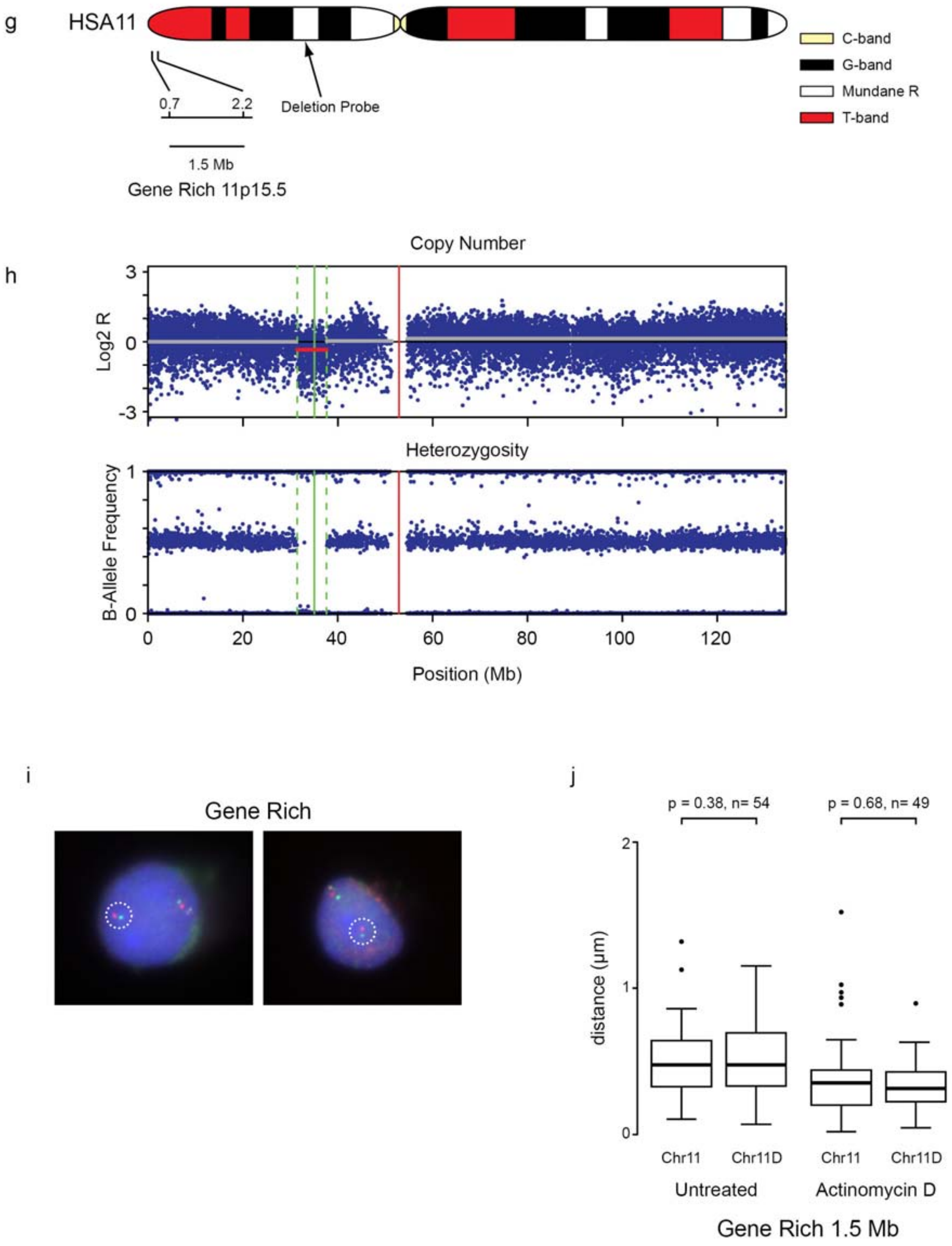

Naughton et al., Supplementary Figure 6

Figure S6. Chromosome X and 11 interphase chromatin fibre compaction in SATO3 and RPE1 cells

This data relates to figure 6. **(a)** SATO cells were cytospun on to slides and fixed with paraformaldehyde. Xist was hybridised using RNA FISH and detected using texas red. Fosmid probes were hybridised using DNA FISH and detected using FITC or Cy5 as detailed in materials and methods. The cells were counterstained with DAPI. The four colour channels were imaged as shown. **(b)**. Location of gene rich Xq13.1 and Xq22.1 regions. **(c)**. 3D DNA/RNA FISH in SATO3 using probes 2 Mb apart (probes are pseudo labelled in green and red and the Xi is marked by a white circle. **(d)**. Distance between probe pairs in individual nuclei. **(e)**. 3D DNA/RNA FISH in RPE1 cells using probes 2 Mb apart (probes are pseudo labelled in green and red and the Xi is marked by a white circle. **(f)**. Distance between probe pairs in individual nuclei. **(g)**. Identification of a gene rich (11p15.5) region on chromosome 11. **(h)**. Mapping chromosome 11 deletion on one allele of the WAGR locus using Illumina 550K SNP arrays. **(i)**. 3D DNA FISH with probes 2 Mb apart flanking the gene rich or gene poor regions and a probe in the WAGR locus. The white circle marks the chromosome 11 carrying the WAGR deletion. **(j)** Distances between pairs of gene rich and gene poor probes before and after transcription inhibition. P values between Chr11 and Chr11Δ are determined by a Wilcoxon test for paired samples, P values before and after transcription inhibition are calculated by a Mann Whitney U test.

Supplemental Experimental Procedures

Cell Culture

SATO3 lymphoblastoid cells were cultured as described previously (Gilbert et al., 2004). Essentially cells were grown in RPMI medium (Invitrogen) supplemented with 10% foetal calf serum, MEM non-essential amino acids (Sigma), 2 mM L-glutamine, 0.5 mM pyruvate 1 mM oxaloacetic acid, 0.2 units/ml human insulin, penicillin/streptomycin and 3 mM MOPS. NIH3T3 cells were gown in DMEM medium (Invitrogen) supplemented with 10% foetal calf serum and penicillin/streptomycin. To inhibit transcription cells were treated with 50 μg/ml actinomycin D for 3 hrs.

Immunofluorescence

Female RPE1 cells were grown on glass slides overnight and female SATO3 cells were grown on poly-L-lysine coated glass slides overnight and processed as described previously (Gilbert et al., 2003). Essentially cells were rinsed with PBS and fixed in either 2% or 4% PFA for 10 min at room temperature. Cells were permeabilised with 0.2% TX100 for 10 min and then blocked in 5% horse serum. Primary antibodies were rabbit anti-MacroH2A (1:100 Upstate 07-219), H3K27me3 (1:100 Upstate 07-449), H3K9me3 (1:500 Upstate 07-442). Secondary antibodies were FITC-conjugated anti-mouse and Texas Red anti-rabbit secondary antibodies (Jackson ImmunoResearch Laboratories).

Live cell imaging

mCherry-macroH2A was constructed from GFP-mCherry (Perche et al., 2000) by replacing GFP with mCherry (Clontech). SATO3 cells were electroporated with plasmid and grown overnight. Live cells were stained with 2 ng/ml Hoechst 33342 for 10 mins, rinsed and embedded in 200 μl agarose matrix (Cygel, Biostatus Ltd) on a Bioptechs imaging dish under a 22 mm glass coverslip (to minimise sample drying). Cells were examined on an inverted Nikon A1R confocal microscope.

Allele specific gene expression

Coding SNPs and their allele frequency were identified from HapMap data on Ensembl and NCBI. SNPs were amplified by touch down PCR using AmpliTaq (Applied Biosystems) from genomic DNA and characterised using Sanger sequencing. To determine which allele genes were expressed from RNA was prepared from SATO3 cells using Tri-Reagent (Sigma), reverse transcribed using Superscript II (Invitrogen), and PCR amplified using AmpliTaq. Samples were then sequenced.

Genomic DNA PCR primers

Gene	SNP	DNA-for	DNA-rev
ATRX	rs3088074	TTCAGCAGAAGGCACAGTTG	CAGTTCCCTTTTTGCTCTGC
ATP7A	rs2227291	GCATAAGACAGCAAATATGGACGTAC	CATAAAAGCATGTATTTCCAATGATTGGC
SH3BGRL	rs1044828	AGAGAAATAACCTGTCTCTCATTCC	GCTTTTGTGCGTGTAAAAGGATATC
GPC4	rs1048369	GCCTGTAATCCCAGCACTTTG	CAAAGAAGTCCACGTCGTTCC
CD99L2	rs6877	GACACTGCCACCTGCAGAAGC	GTCCCAGAGCCTCCCCTCTG
XIST	rs1620574	GCTAGGCATTGGGGATGCAAAG	GGCATGTAGTTCCGAGCCCC

cDNA PCR primers

Gene	SNP	RNA-for	RNA-rev
ATRX	rs3088074	same as DNA	same as DNA
ATP7A	rs2227291	same as DNA	CTGAGGAGGATATTATCAGAATCAAGAG
SH3BGRL	rs1044828	same as DNA	same as DNA
GPC4	rs1048369	GCCAAGAAATTCTGGTCCTCC	same as DNA
CD99L2	rs6877	same as DNA	same as DNA
XIST	rs1620574	same as DNA	same as DNA

Chromatin Fractionation and Microarray Hybridisation

Nuclei from cells were prepared as described previously (Gilbert et al., 2004). Nuclei were digested with micrococcal nuclease and soluble chromatin was released by gentle lysis. Soluble chromatin was fractionated on a 6-40% isokinetic sucrose gradient in 80 mM NaCl, 0.1 mM EDTA, 0.1 mM EGTA and 250 µM PMSF.

For “open” chromatin analysis samples were processed as described previously (Gilbert et al., 2004). The DNA from a single gradient fraction was purified and fractionated on a pulsed field gel. DNA of 18 – 20 Kbp was isolated corresponding to “open” chromatin whilst bulk chromatin was approximately 10.5 Kbp. For microarray analysis DNA was isolated from gel slices using electroelution and amplified by whole genome amplification (Sigma). Amplified DNA was hybridised to Illumina arrays according to the manufacturers instructions.

Genotyping

For genotyping genomic DNA was prepared from cell lines by phenol/chloroform extraction and hybridised to Illumina SNP arrays according to the manufacturer.

Gene expression data

RNA was prepared from SATO3 (female lymphoblastoid) and FATO (male lymphoblastoid) cells in triplicate using Tri-Reagent (Sigma) extraction and isopropanol precipitation. The RNA was resuspended in nuclease-free water and subjected to further phenol/chloroform extraction and precipitation. The RNA integrity and concentration were analysed on an Agilent Bioanalyzer. cRNA was prepared using an Illumina TotalPrep RNA amplification kit (Ambion) according to the instructions. RNA was hybridised to Illumina HT-12 or Affymetrix U133A arrays. Array normalisation and analysis were carried out using dChip or the lumi bioconductor package (Du et al., 2008). SAGE data was obtained from the Cancer Genome Atlas Project. To approximate lymphoblastoid cells we used a SAGE library generated from monocyte depleted mononuclear cells (GSM384027 and GSM384028) (Patino et al., 2005). Tissue and cell line expression array data was also obtained from the Genomics Institute of the Novartis Research Foundation (GNF) data sets (http://biogps.gnf.org). The upstream and downstream positions of DNaseI hypersensitive sites were taken from (Boyle et al., 2008).

3D RNA/DNA Four Color FISH

Non-adherent lymphoblastoid cells (2 x 10^4 cells) were cytospun on to glass slides at 600 rpm for 10 min. Slides were rinsed with PBS and fixed in 4% paraformaldehyde, 10 minutes at room temperature. Slides were rinsed with PBS and cells were permeabilised for 10 minutes on ice with PBS/0.2% triton /2mM Vanadyl Ribonuclease Complex. After rinsing with PBS slides were stored in 70% EtOH at 4°C.

For processing, slides were dehydrated through an ethanol series and air-dried before probe hybridisation at 37°C overnight. 200ng of digoxigenin-11-dUTP (Roche) labelled XIST probe (a gift from Laura Carrel, Penn State) was hybridised with 5μg Salmon Sperm DNA and 20μg tRNA. Slides were washed: 1× 3 mins 50% formamide/2×SSC at room temperature, 1× 3 mins 50%formamide/2×SSC at 37°C, 1× 3 mins 2×SSC at room temperature, 1× 3 mins 4×SSC/0.1 Tween 20 at room temperature. Digoxigenin-labelled probes were detected by using rhodamine anti-digoxigenin and Texas red anti-sheep IgG (Vector Laboratories). For subsequent DNA FISH The RNA signal was fixed in 4% PFA for 30 minutes followed by denaturation in 50%formamide/2×SSC at 80°C for 30 minutes. Slides were then dipped briefly in 2×SSC at room temperature before overnight hybridisation at 37°C with pairs of probes (listed in supplementary materials and methods) labeled in digoxigenin-11-dUTP or biotin-16-dUTP. 150ng of each labelled probe was hybridised with 5μg of Salmon Sperm and 10μg of human Cot1 DNA. Slides were washed: 4× 3 mins 2×SSC at 45°C, 4× 3 mins 0.1×SSC at 60°C before transferring to 4×SSC/0.1% Tween 20 at room temperature. Digoxigenin-labeled probes were detected by using one layer of rhodamine conjugated sheep anti-digoxigenin and a second layer of Texas red conjugated anti-sheep (Vector Laboratories). Biotin-labeled probes were detected by using one layer of biotin conjugated anti-avidin and a second

layer of Cy5 conjugated streptavidin (Vector Laboratories). Slides were counterstained with 0.5μg/ml DAPI.

Image Capture and Analysis

Four colour 3D RNA/DNA FISH slides were imaged using a Hamamatsu Orca AG CCD camera (Hamamatsu Photonics (UK) Ltd, Welwyn Garden City, UK) Zeiss Axioplan II fluorescence microscope with Plan-neofluar objectives, a 100W Hg source (Carl Zeiss, Welwyn Garden City, UK) and Chroma #86000v2 quadruple band pass filter set (Chroma Technology Corp., Rockingham, VT) with the single excitation and emission filters installed in motorised filter wheels (Prior Scientific Instruments, Cambridge, UK). Image capture and analysis were performed using in-house scripts written for IPLab Spectrum (Scanalytics Corp, Fairfax, VA). For three colour imaging the microscope was the same but a Chroma #83000 triple band pass filter set was used with single excitation filters installed. For FISH, images were collected from at least 50 randomly selected nuclei for each experiment and then analyzed by using custom IPLab scripts that calculate either the distance between two probe signals or the nuclear localisation of the chromosome territory. The statistical significance of compaction between the Xa and the Xi was tested using the nonparametric Wilcoxon test for paired samples whilst p values between chromosomal loci was calculated by using the nonparametric Mann–Whitney U test (using R programming). $P < 0.05$ has been taken as statistically significant. For color images shown in the paper the gamma values have been adjusted to enhance the appearance of the image. In addition, the “blue” colour has been adjusted by adding additional RED and GREEN signal to the relevant channels to lighten the blue colour.

Clonality Assay

To assess the clonality of X-inactivation in SATO clones we PCR amplified a variable CAG repeat in the non-coding exon 1 of the human androgen receptor located on the X chromosome (Shattuck et al., 2005). EcoRI digested genomic

DNA prepared from the cells was digested in the presence or absence of HpaII and subject to PCR using primers 5'-TCTGTTCCAGAGCGTGCGCGAAGT-3' and TET-5'-CTCTACGATGGGCTTGGGGAGAAC-3'. DNA samples were purified and analysed on an ABI3730 capillary sequencer with ROX labelled 400HD markers. The data was analysed and quantified using ABI GeneMapper Software v3.7.

Mapping heterozygous deletion on chromosome 11

To distinguish between the homologous chromosome 11s we took advantage of a heterozygous deletion at 11p13 in the WAGR locus (van Heyningen et al., 1985). SATO3 cells were genotyped using standard clustering of SNPs from Illumina's Bead Studio (Version 3.13) software to map the deletion to identify copy number changes and loss of heterozygosity at the locus (Supplementary Fig. 6h). Hybridisation of a fosmid probe labelled in both FITC and Cy5 located in the deletion region enabled us to reproducibly distinguish the normal chromosome 11 and the chromosome 11 carrying the deletion (Supplementary Fig. 6i).

Mapping chromosome X and 7 haplotypes

SATO3 cells carry two X chromosomes. We obtained a lymphoblastoid cell line (FATO) from the father of the individual that SATO3 cells was isolated from. It carries one of the X-chromosomes and the other SATO3 X-chromosome was transferred to a mouse-human hybrid (SAX1)(Porteous et al., 1987). The mouse human hybrid also carries single copies of some autosomes, including chromsosome 7. All cell lines were genotyped by hybridising to Illumina Human HapMap 550K Genotyping SNP Arrays. SATO3 and FATO cells were genotyped using standard clustering of SNPs from Illumina's Bead Studio (Version 3.13) software.

The haplotypes of each of the two copies of the X chromosome in SATO3 cells were determined in two stages. Firstly, 3 hybridisations of SATO genomic DNA

to the SNP arrays were used to determine the genotype of each SNP on the X chromosome. SATO SNPs were called as heterozygous or homozygous if at least two of these hybridisations agreed. Around 4000 X chromosome SNPs were determined to be heterozygous in SATO cells and therefore, informative for our chromatin assay. The separate alleles of these heterozygous SNPs were then assigned to either the maternal or paternal X chromosome by examining genotyping data from the FATO cell line which carries a single copy of the paternal X chromosome from SATO cells (FATO cells are a lymphoblastoid cell line from the father of the individual from which SATO cells were isolated). The genotype of the FATO X chromosome SNPs was determined in a similar manner using two hybridisations.

The genotyping of the two homologues of chromosome 7 in SATO cells was more complex. DNA isolated from the mouse-human hybrid cell line SAX1 created from SATO3 cells was hybridised to SNP arrays. In SAX1 both homologues of some of the chromosomes, including chromosome 7, are present in unequal quantities in the hybrid. The standard Illumina genotyping methods rely on the comparison of a single sample to a 'bank' of normal samples performed by Illumina. However, it has been observed that the standard Illumina protocols induce biases in the data when dealing with highly 'abnormal' samples with a large variation in copy number, such as the SAX1 hybrid cell line (data not shown and (Staaf et al., 2008)). Therefore, this data was analysed by treating SATO3 and SAX1 as a pair. Briefly, the raw mean intensity of each beadset (ie each SNP) and allele type (X and Y channels) was exported from Illumina BeadStudio and analysed in R. Paired analyses of Illumina SNP samples have been observed to provide a better way to analyse non-standard samples (Peiffer et al., 2006). So, using R and the Bioconductor package limma, the raw X and Y intensities for each SNP (representing the two alleles) were quantile normalised to each other. Then for each SNP a ratio of the normalised Y/X was calculated. The ratio observed in SATO3 cells represents the ratio we expect for that SNP in the heterozygous state (bearing in mind that at this point we are only looking at

SNPs that are heterozygous in SATO cells). If the ratio of Y/X in SAX1 is greater than this value then the most abundant chromosomal homologue in SAX1 carries a B allele and if the ratio is lower it carries the A allele. To carry out this genotyping in an automatic fashion the ratio of SAX1 Y/X over SATO Y/X was calculated and subject to the same Theta transformation used to generate the B-allele frequency in CGH experiments using Illumina SNP arrays (Peiffer et al., 2006). For SATO3 heterozygous SNPs on each chromosome this produced a bi-modal distribution of transformed ratios. The density minimum between these two peaks was found and SNPs with a ratio greater than this value assigned as B in the most abundant SAX1 homologue and those with a ratio less than this value assigned A.

SNP array data processing

SNP Array Data was processed using the R statistical software and the limma Bioconductor Package. SNP array data was extracted using Illumina Beadstudio and the raw mean bead intensities exported for each SNP probe. Exported data were loaded into R and normalised using limma and block loess normalisation. SNP arrays were analysed in a paired sample manner comparing open to input samples. For most analyses the mean of two replicate hybridisations was used but results were checked for consistency between the two replicates. All analyses were performed using standard statistical functions within R. Gene positions were downloaded from Ensembl (version 53 or 54). Chromosome banding positions were taken from the UCSC browser (May 2006, hg18). The positions of the XAR and XCR were taken from (Wang et al., 2006) and converted from hg17 to hg18 using the UCSC browser's convert tool. The positions of the PAR regions and XTR were defined as midway between the first and last genes of each region, the genes were taken from "The DNA Sequence of the Human X Chromosome" Nature 2005 and positions defined in hg18. Running means were calculated using a script in R.

Cosmid and Fosmid Probes

(All positions are NCBI36/hg18 assembly)

Chromosome X – Gene Rich

G248P89772C1	Xq13.1	68,999,544 – 69,041,544
G248P87866A9	Xq13.1	70,987,645 – 71,024,210
G248P83958D6	Xq13.1	71,480,003 – 71,519,299
G248P87670C3	Xq22.1	100,040297 – 100,084,987
G248P83908C7	Xq22.1	101,977,866 – 102,020,280

Chromosome X – Gene Poor

G248P88902B4	Xq24	120,016,869 – 120,055,863
G248P8676F2	Xq25	121,975,898 – 122,015,644

Chromosome 11 – Gene Poor

G248P87052F5	11p14.1	28048686 – 28089211
G248P86663E9	11p14 .1	30041976 – 30082407

Chromosome 11 – Gene Rich

cl11p15-49	11p15.5	736369 – 755546
h19igf2	11p15.5	≈2100000 - 2120000

Chromosome 11 deletion probe

G248P8419H11	11p13	35044927 – 35045591

Supplemental References

Boyle,A.P., Davis,S., Shulha,H.P., Meltzer,P., Margulies,E.H., Weng,Z., Furey,T.S., and Crawford,G.E. (2008). High-resolution mapping and characterization of open chromatin across the genome. Cell. *132*, 311-322.

Du,P., Kibbe,W.A., and Lin,S.M. (2008). lumi: a pipeline for processing Illumina microarray. Bioinformatics. *24*, 1547-1548.

Gilbert,N. and Allan,J. (2001). Distinctive higher-order chromatin structure at mammalian centromeres. Proc. Natl. Acad. Sci. U. S. A *98*, 11949-11954.

Gilbert,N., Boyle,S., Fiegler,H., Woodfine,K., Carter,N.P., and Bickmore,W.A. (2004). Chromatin architecture of the human genome: gene-rich domains are enriched in open chromatin fibers. Cell *118*, 555-566.

Gilbert,N., Boyle,S., Sutherland,H., de Las,H.J., Allan,J., Jenuwein,T., and Bickmore,W.A. (2003). Formation of facultative heterochromatin in the absence of HP1. EMBO J. *22*, 5540-5550.

Harrison,B.D. and Klug,A. (1966). Relation between length and sedimentation coefficient for particles of tobacco rattle viruses. Virology *30*, 738-740.

Hartley,P.D. and Madhani,H.D. (2009). Mechanisms that specify promoter nucleosome location and identity. Cell. *137*, 445-458.

Jin,C., Zang,C., Wei,G., Cui,K., Peng,W., Zhao,K., and Felsenfeld,G. (2009). H3.3/H2A.Z double variant-containing nucleosomes mark 'nucleosome-free regions' of active promoters and other regulatory regions. Nat. Genet. *41*, 941-945.

Patino,W.D., Mian,O.Y., Kang,J.G., Matoba,S., Bartlett,L.D., Holbrook,B., Trout,H.H., III, Kozloff,L., and Hwang,P.M. (2005). Circulating transcriptome reveals markers of atherosclerosis. Proc. Natl. Acad. Sci. U. S. A. *102*, 3423-3428.

Peiffer,D.A., Le,J.M., Steemers,F.J., Chang,W., Jenniges,T., Garcia,F., Haden,K., Li,J., Shaw,C.A., Belmont,J., Cheung,S.W., Shen,R.M., Barker,D.L., and Gunderson,K.L. (2006). High-resolution genomic profiling of chromosomal aberrations using Infinium whole-genome genotyping. Genome Res. *16*, 1136-1148.

Perche,P.Y., Vourc'h,C., Konecny,L., Souchier,C., Robert-Nicoud,M., Dimitrov,S., and Khochbin,S. (2000). Higher concentrations of histone macroH2A in the Barr body are correlated with higher nucleosome density. Curr. Biol. *10*, 1531-1534.

Porteous,D.J., Bickmore,W., Christie,S., Boyd,P.A., Cranston,G., Fletcher,J.M., Gosden,J.R., Rout,D., Seawright,A., Simola,K.O., and . (1987). HRAS1-selected chromosome transfer generates markers that colocalize aniridia- and genitourinary dysplasia-associated translocation breakpoints and the Wilms tumor gene within band 11p13. Proc. Natl. Acad. Sci. U. S. A. *84*, 5355-5359.

Schones,D.E., Cui,K., Cuddapah,S., Roh,T.Y., Barski,A., Wang,Z., Wei,G., and Zhao,K. (2008). Dynamic regulation of nucleosome positioning in the human genome. Cell. *132*, 887-898.

Shattuck,T.M., Westra,W.H., Ladenson,P.W., and Arnold,A. (2005). Independent clonal origins of distinct tumor foci in multifocal papillary thyroid carcinoma. N. Engl. J. Med. *352*, 2406-2412.

Staaf,J., Vallon-Christersson,J., Lindgren,D., Juliusson,G., Rosenquist,R., Hoglund,M., Borg,A., and Ringner,M. (2008). Normalization of Illumina Infinium whole-genome SNP data improves copy number estimates and allelic intensity ratios. BMC. Bioinformatics. *9:409.*, 409.

van Heyningen,V., Boyd,P.A., Seawright,A., Fletcher,J.M., Fantes,J.A., Buckton,K.E., Spowart,G., Porteous,D.J., Hill,R.E., Newton,M.S., and . (1985). Molecular analysis of chromosome 11 deletions in aniridia-Wilms tumor syndrome. Proc. Natl. Acad. Sci. U. S. A. *82*, 8592-8596.

Wang,Z., Willard,H.F., Mukherjee,S., and Furey,T.S. (2006). Evidence of influence of genomic DNA sequence on human X chromosome inactivation. PLoS. Comput. Biol. *2*, e113.
